# Skeletal muscle mitochondrial fragmentation predicts age‐associated decline in physical capacity

**DOI:** 10.1111/acel.14386

**Published:** 2024-12-04

**Authors:** Richie P. Goulding, Braeden T. Charlton, Ellen A. Breedveld, Matthijs van der Laan, Anne R. Strating, Wendy Noort, Aryna Kolodyazhna, Brent Appelman, Michèle van Vugt, Anita E. Grootemaat, Nicole N. van der Wel, Jos J. de Koning, Frank W. Bloemers, Rob C. I. Wüst

**Affiliations:** ^1^ Department of Human Movement Sciences, Faculty of Behavioural and Movement Sciences, Amsterdam Movement Sciences Vrije Universiteit Amsterdam Amsterdam The Netherlands; ^2^ Center for Experimental and Molecular Medicine Amsterdam UMC Location University of Amsterdam Amsterdam The Netherlands; ^3^ Amsterdam Institute for Infection and Immunity Amsterdam The Netherlands; ^4^ Division of Infectious Diseases, Tropical Medicine, Department of Medicine Amsterdam UMC Location University of Amsterdam Amsterdam The Netherlands; ^5^ Electron Microscopy Centre Amsterdam Amsterdam UMC Location Academic Medical Centre Amsterdam The Netherlands; ^6^ Department of Trauma Surgery, Amsterdam Movement Sciences, Amsterdam UMC Vrije Universiteit Amsterdam Amsterdam The Netherlands

**Keywords:** ageing, maximal oxygen uptake, mitochondrial morphology, mitochondrial respiration, skeletal muscle

## Abstract

Ageing substantially impairs skeletal muscle metabolic and physical function. Skeletal muscle mitochondrial health is also impaired with ageing, but the role of skeletal muscle mitochondrial fragmentation in age‐related functional decline remains imprecisely characterized. Here, using a cross‐sectional study design, we performed a detailed comparison of skeletal muscle mitochondrial characteristics in relation to in vivo markers of exercise capacity between young and middle‐aged individuals. Despite similar overall oxidative phosphorylation capacity (young: 99 ± 17 vs. middle‐aged: 99 ± 27 pmol O_2_.s^−1^.mg^−1^, *p* = 0.95) and intermyofibrillar mitochondrial density (young: 5.86 ± 0.57 vs. middle‐aged: 5.68 ± 1.48%, *p* = 0.25), older participants displayed a more fragmented intermyofibrillar mitochondrial network (young: 1.15 ± 0.17 vs. middle‐aged: 1.55 ± 0.15 A.U., *p* < 0.0001), a lower mitochondrial cristae density (young: 23.40 ± 7.12 vs. middle‐aged: 13.55 ± 4.10%, *p* = 0.002) and a reduced subsarcolemmal mitochondrial density (young: 22.39 ± 6.50 vs. middle‐aged: 13.92 ± 4.95%, *p* = 0.005). Linear regression analysis showed that 87% of the variance associated with maximal oxygen uptake could be explained by skeletal muscle mitochondrial fragmentation and cristae density alone, whereas subsarcolemmal mitochondrial density was positively associated with the capacity for oxygen extraction during exercise. Intramuscular lipid accumulation was positively associated with mitochondrial fragmentation and negatively associated with cristae density. Collectively, our work highlights the critical role of skeletal muscle mitochondria in age‐associated declines in physical function.

## Graphical abstract

When comparing young versus middle‐aged individuals, we found that older individuals possessed a reduced skeletal muscle subsarcolemmal mitochondrial density, a more fragmented intermyofibrillar mitochondrial network and lower mitochondrial cristae density. These mitochondrial alterations were strongly associated with physical function across groups, implying an important role of mitochondria in age‐related skeletal muscle decline.

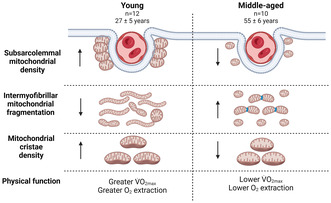

**Abbreviations**
$\dot{V}$O_2max_
: maximal oxygen uptake
A.U.: arbitrary units
ATP: adenosine triphosphate
BMI: body mass index
CPET: cardiopulmonary exercise testing
DmO_2_
: diffusing capacity for oxygen
ETS: Electron transport system
FCCP: carbonylcyanide‐p‐trifluoromethoxyphenylhydrazone
JO_2_
: mitochondrial oxygen consumption
MFI: mitochondrial fragmentation index
MICOS: mitochondrial contact site and cristae organizing system
NIRS: near‐infrared spectroscopy
OXPHOS: oxidative phosphorylation capacity
RBC: red blood cell
ROT: rotenone
SD: standard deviation
SDH: succinate dehydrogenase
TEM: transmission electron microscopy
UEA‐1: Ulex Europaeus Agglutin 1
VIF: variance inflation factor

## 1 INTRODUCTION

Within the last century, considerable advances in healthcare have resulted in a worldwide demographic shift toward an ageing population (Salomon et al., 2012). This demographic shift has not been paralleled by a commensurate shift in health span, and thus the burden of the impaired physical function and age‐related comorbidities on healthcare systems has increased markedly (Butler et al., 2008). A better understanding of the biological processes of ageing is therefore a scientific necessity in order to counter age‐related pathologies and health decline.

Skeletal muscle is an organ that is strikingly affected by ageing (Tieland et al., 2018), and the loss of skeletal muscle health and function that occurs with old age is causally implicated in the reduced mobility (Tian et al., 2022), increased risk of falls (Gadelha et al., 2018), physical frailty (Fried et al., 2001) and inability to complete tasks of daily living (Tieland et al., 2018) that occurs with age. One hallmark of biological ageing is mitochondrial dysfunction (López‐Otín et al., 2013), which occurs with ageing across various organ systems (Green et al., 2011), including skeletal muscle (Gouspillou et al., 2014) and age‐related mitochondrial dysfunction is strongly associated with impaired physical function (Grevendonk et al., 2021). Skeletal muscle mitochondria exist within distinct subpopulations, including the subsarcolemmal and the intermyofibrillar mitochondria, which together form an interconnected reticulum (Glancy et al., 2015). The subsarcolemmal mitochondria located closer to the capillaries generate and transduce the protonmotive force towards the intermyofibrillar mitochondria, where energetic demand and ATP production are greatest (Glancy et al., 2015). Mitochondrial fragmentation impairs this energy transduction, but can equally prevent widespread transduction of mitochondrial damage across the reticulum (Glancy et al., 2017). Excessive mitochondrial fission and fragmentation causes rapid muscle wasting in mice (Romanello et al., 2010), whereas increasing mitochondrial fusion leads to hypertrophy (Cefis et al., 2024), suggesting a link between mitochondrial fragmentation and sarcopenia. We have recently demonstrated that mitochondrial fragmentation occurs after 6 days of bed rest, prior to the reduction in skeletal muscle oxidative phosphorylation capacity which occurred after 55 days (Eggelbusch et al., 2024). However, whether skeletal muscle mitochondrial fragmentation is an early marker for human ageing before overt sarcopenia occurs, and whether this process is linked with impaired whole‐body physical function, remains unknown.

Here, we characterized skeletal muscle mitochondrial characteristics in young and middle‐aged humans in order to determine whether differences in mitochondrial fragmentation were related to physical function. We studied active middle‐aged individuals in order to exclude that age‐related differences were due to physical inactivity or sarcopenia per se, and hypothesized that mitochondrial fragmentation could be an early‐ageing phenotype that precedes significant loss of skeletal muscle mass and physical function. We show that, independent of mitochondrial oxidative phosphorylation capacity or intermyofibrillar mitochondrial area density, middle‐aged individuals displayed a more fragmented mitochondrial network, a lower subsarcolemmal mitochondrial area density and reduced mitochondrial cristae area density. Mitochondrial cristae density and mitochondrial fragmentation strongly predicted whole‐body physical capacity, whereas subsarcolemmal mitochondrial density was closely associated with muscle O_2_ extraction capacity. As such, alterations in intramuscular mitochondrial networks are an early marker for the age‐associated changes in physical capacity.

## 2 RESULTS

### 2.1 Reduced exercise capacity in middle‐age is linked to peripheral O_2_ extraction

**FIGURE 1:**
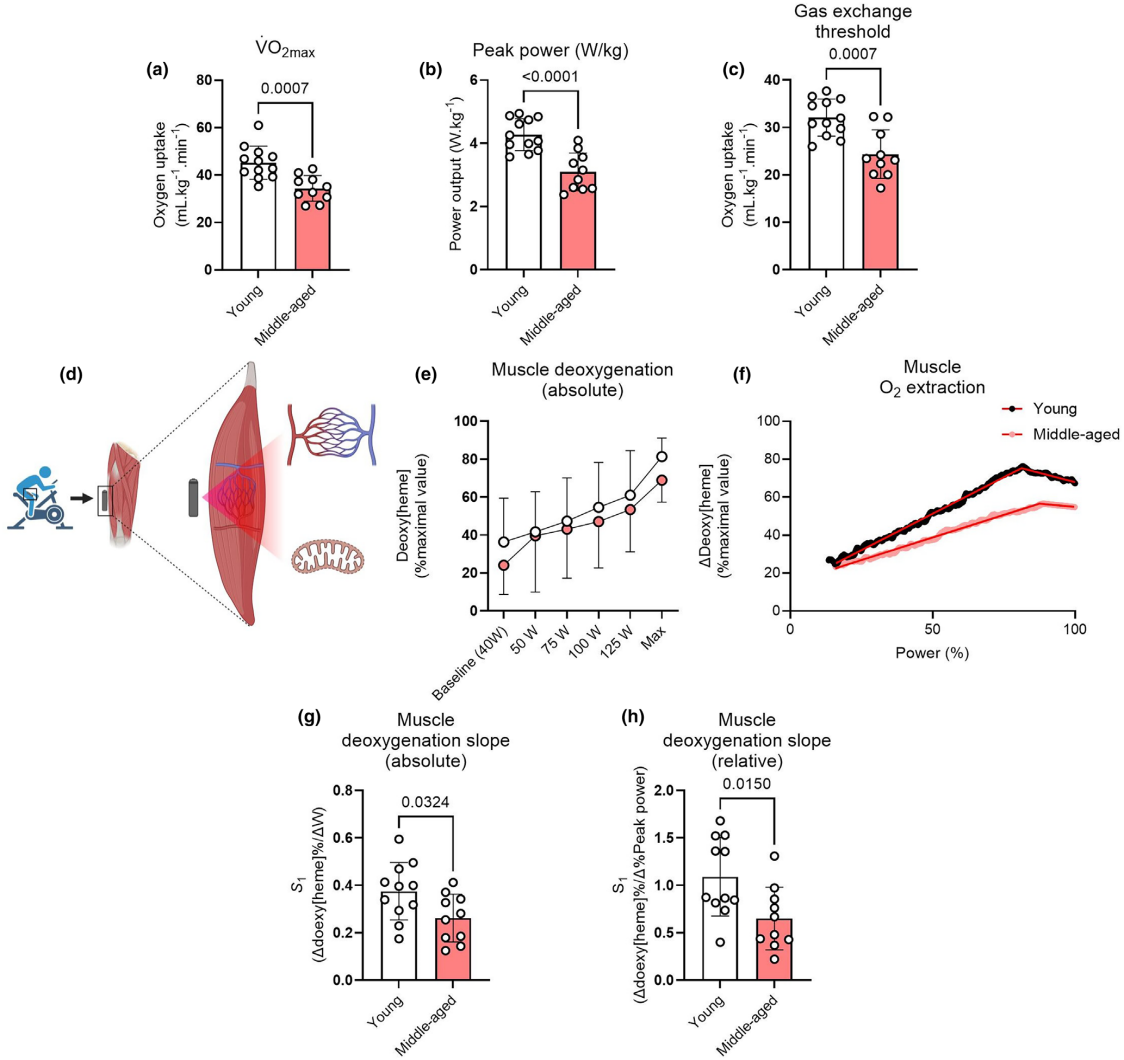
(a), maximal pulmonary oxygen uptake ($\dot{V}$O_2max_); (b), peak power normalized by body mass; (c), gas exchange threshold. (d), near‐infrared spectroscopy (NIRS) was employed to noninvasively monitor muscle deoxygenation during exercise. (e), group mean ± SD muscle deoxygenation versus absolute power output in young (clear) and middle‐aged (pink) groups. (f), representative muscle deoxygenation responses to exercise in both groups with modelled fits (solid red lines). (g) and (h), the initial slope of muscle deoxygenation versus work rate during exercise as a function of absolute and relative power, respectively. Circles reflect individual data points for each participant. Columns and error bars reflect means ± SD. *p* values displayed above each panel.

First, we determined exercise capacity in 12 young (mean age 27 ± 5 years) and 10 middle‐aged (55 ± 6 years) participants using an incremental cardiopulmonary exercise test (Table 1). Participants were included if they were recreationally physically active (Table S1). Maximal oxygen uptake ($\dot{V}$O_2max_, Figure 1a) and peak power output normalized by body mass (Figure 1b), key markers of exercise capacity and age‐related physical decline, were lower in the middle‐aged compared to the young participants. The gas exchange threshold, a submaximal index of the capacity to maintain whole‐body blood acid–base homeostasis during submaximal exercise, was also lower in middle‐aged compared to young participants (Figure 1c). Aside from the expected age‐associated reduction in maximal heart rate, there was a notable lack of differences in cardiovascular or pulmonary function throughout the exercise test between groups (Table S1), implying that peripheral skeletal muscle impairments in the middle‐aged participants were likely contributory to the reduced exercise capacity.

**TABLE 1:** Cohort characteristics (values displayed are mean ± SD).

	Young	Middle‐aged	*p* Value
*n*	12	10	
Sex (male/female)	8/4	5/5	0.43
Age (years)	27 ± 5	55 ± 6	<0.001^a^
Height (cm)	178 ± 8	177 ± 7	0.70
Weight (kg)	70 ± 13	79 ± 14	0.11
BMI (kg/m^2^)	22 ± 2	25 ± 3	0.017^a^

^a^ Significant difference between groups (*p* < 0.05).

To provide further insight into potential peripheral contributions to reduced exercise tolerance, we monitored muscle O_2_ extraction noninvasively throughout the exercise test using near‐infrared spectroscopy (NIRS) (Figure 1d,e). The muscle deoxygenation responses to incremental exercise were fitted with a double‐linear function in order to characterize the degree of reliance on O_2_ extraction during exercise (Figure 1f). Indeed, muscle deoxygenation was reduced in the middle‐aged compared to the young group (Figure 1f–h, Figure S1), and the initial slope of muscle deoxygenation was strongly correlated with $\dot{V}$O_2max_ across groups (Figure S2), indicating a role for impaired O_2_ extraction in the reduced $\dot{V}$O_2max_ in middle‐age.

We reasoned that, given the age‐associated alterations in exercising blood flow (Musch et al., 2004; Proctor et al., 1998), greater blood flow relative to local $\dot{V}$O_2_ (i.e., enhanced $\dot{Q}$/$\dot{V}$O_2_) was unlikely to explain the blunted deoxygenation we observed in middle‐aged subjects. Rather, a reduction in muscle diffusing capacity for O_2_ (DmO_2_) could contribute (Roca et al., 1992). DmO_2_ is largely determined by the aggregate number of red blood cells (RBCs) in apposition to the contracting myocytes (Federspiel & Popel, 1986), which may be indirectly inferred via changes in total [heme] during exercise measured via NIRS. The increase in total [heme] from rest to maximal exercise was larger in middle‐aged compared to young participants (Figure S1B,C), and correlated with $\dot{V}$O_2max_ in young but not middle‐aged participants (Figure S2C).

DmO_2_ is also influenced by structural variables related to capillarization and mitochondrial metabolism (Hepple et al., 2000). To this end, we obtained skeletal muscle biopsies from the vastus lateralis in all participants and assessed markers of capillarization and mitochondrial enzyme activity (Figure S3). The mean fibre cross‐sectional area, muscle capillary density and the capillary‐to‐fibre ratio did not differ between groups (Figure S3A–E), implying that the age‐related impairments in exercise capacity and O_2_ extraction were not the result of alterations in capillarization. Conversely, muscle succinate dehydrogenase (SDH) activity was lower in middle‐aged individuals, an effect that was driven primarily by lower SDH activity in highly oxidative, rather than low oxidative fibres (Figure S3F–J). These findings indicate that a reduced peripheral O_2_ extraction contributes to reduced physical function in middle‐aged humans, and that this may be related to skeletal muscle mitochondrial alterations or altered capillary [hematocrit] changes upon exercise, rather than impaired structural indices of capillarization.

### 2.2 Skeletal muscle mitochondrial fragmentation in ageing

**FIGURE 2:**
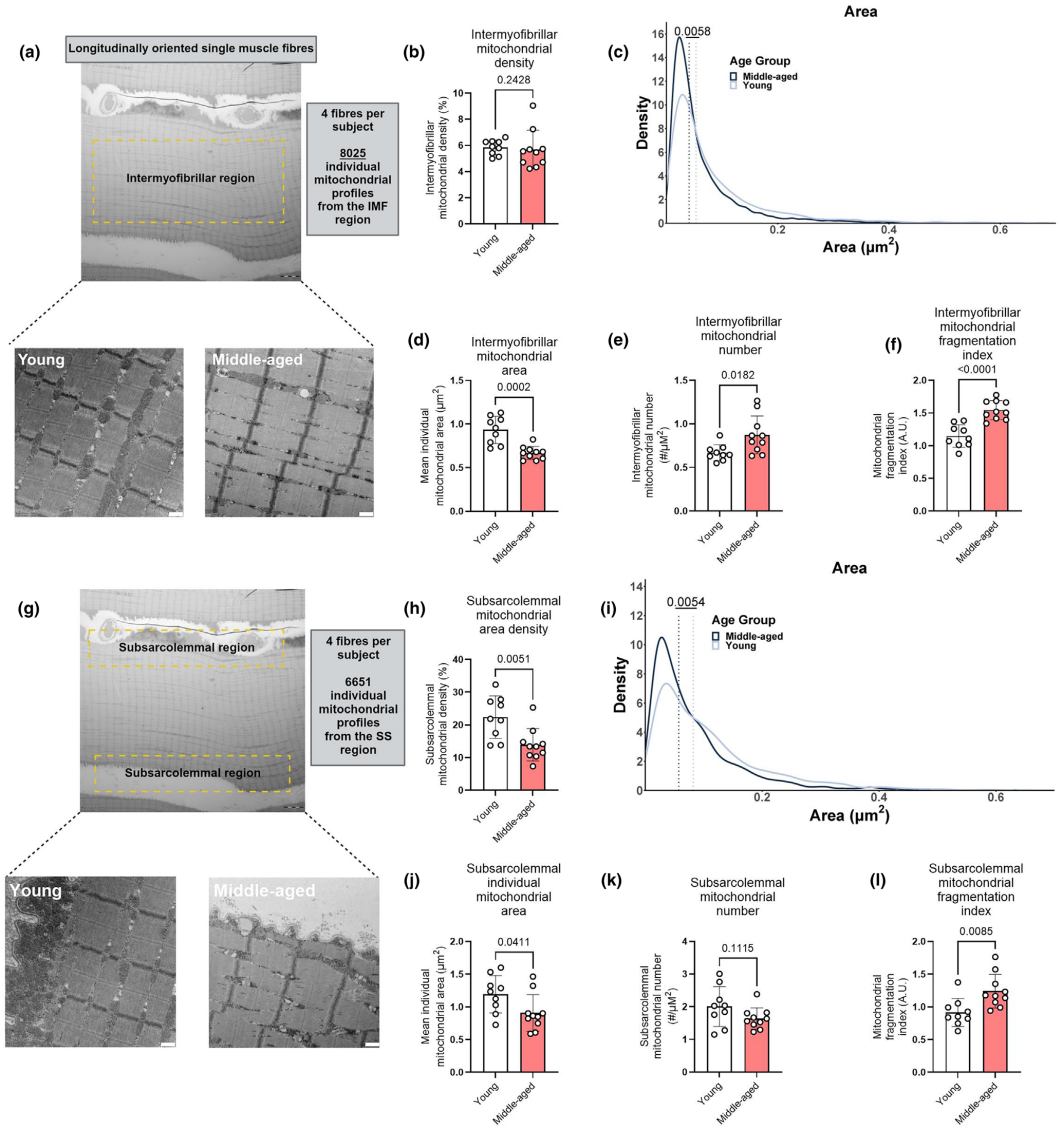
(a), representative transmission electron microscopy images used for quantification in intermyofibrillar mitochondrial analysis (scale bar = 500 nm). (b), intermyofibrillar mitochondrial density; (c), frequency distribution of individual intermyofibrillar mitochondrial areas in both groups; (d), mean individual intermyofibrillar mitochondrial area; (e), intermyofibrillar mitochondrial number per μm^2^; (f), intermyofibrillar mitochondrial fragmentation index. (g), representative images used for quantification in subsarcolemmal mitochondrial analysis (scale bar = 500 nm). (h), subsarcolemmal mitochondrial density; (i), frequency distribution of individual subsarcolemmal mitochondrial areas in both groups; (j), mean individual subsarcolemmal mitochondrial area; (k), subsarcolemmal mitochondrial number per μm^2^; (l), subsarcolemmal mitochondrial fragmentation index. Circles reflect individual data points for each participant. Columns and error bars reflect means ± SD. *p* values are displayed above each panel. Dashed lines of plots (c) and (i) reflect the median values of each distribution.

As our findings indicated that reduced physical capacity and O_2_ extraction were linked to intramuscular mitochondrial alterations, we next sought to examine whether morphological or ultrastructural skeletal muscle mitochondrial alterations exist between young and middle‐aged humans. We therefore performed transmission electron microscopy on single skeletal muscle fibres (Figure 2a), and imaged both the intermyofibrillar and subsarcolemmal region. Within the intermyofibrillar region, a total of 8025 individual mitochondrial profiles were manually traced to determine density and morphology (i.e., 15 ± 2 images of the intermyofibrillar region per participant; Figure 2a). Intermyofibrillar mitochondrial density did not differ between groups (Figure 2b), however, middle‐aged subjects possessed smaller (Figure 2c,d) yet more numerous mitochondria (Figure 2c,e). The mitochondrial fragmentation index provides an indication of the degree of fragmentation of the mitochondrial pool (Gemmink et al., 2023), and this was also greater in middle‐aged compared to younger individuals (Figure 2f). Morphological size markers (including perimeter, maximal and minimal Feret's diameter and perimeter‐to‐area ratio) were lower in the middle‐aged versus the young group, whereas mitochondrial shape markers (circularity, roundness, aspect ratio) did not differ between the groups (Figure S4). Collectively, these findings demonstrate that middle‐aged individuals possess a fragmented intermyofibrillar mitochondrial network, consisting of smaller and more numerous mitochondria. Given previous research highlighting the functional connectivity of the skeletal muscle mitochondrial reticulum (Glancy et al., 2015, 2017), we hypothesized that this mitochondrial fragmentation could contribute to reduced physical function with ageing.

Compared to the intermyofibrillar mitochondria, the subsarcolemmal mitochondria display greater network connectivity, increased electron transport system (ETS) conductance and are responsible for transducing the protonmotive force towards the core of the myofibres where energetic demand is greatest (Glancy et al., 2015, 2017; Parry et al., 2024). Hence, we expected that alterations in subsarcolemmal mitochondrial content or morphology would contribute to the age‐associated reduction in physical capacity. We therefore assessed mitochondrial density and morphology in the subsarcolemmal region using transmission electron microscopy (Figure 2g). We manually traced 6651 individual mitochondrial profiles (from 13 ± 3 images/participant). In contrast to the intermyofibrillar region, middle‐aged participants displayed a lower subsarcolemmal mitochondrial density (Figure 2h), primarily driven by a smaller area of individual mitochondria (Figure 2i,j), rather than fewer mitochondria per μm^2^ (Figure 2i,k). The mitochondrial fragmentation index was also greater in middle‐aged compared to young individuals (Figure 2l). Mitochondrial size markers were lower in the middle‐aged group, with no differences in mitochondrial shape markers (Figure S5). Collectively, these findings indicate that the intermyofibrillar and subsarcolemmal mitochondrial subpopulations within the mitochondrial reticulum are differentially affected by ageing.

### 2.3 Mitochondrial cristae density and respiration

**FIGURE 3:**
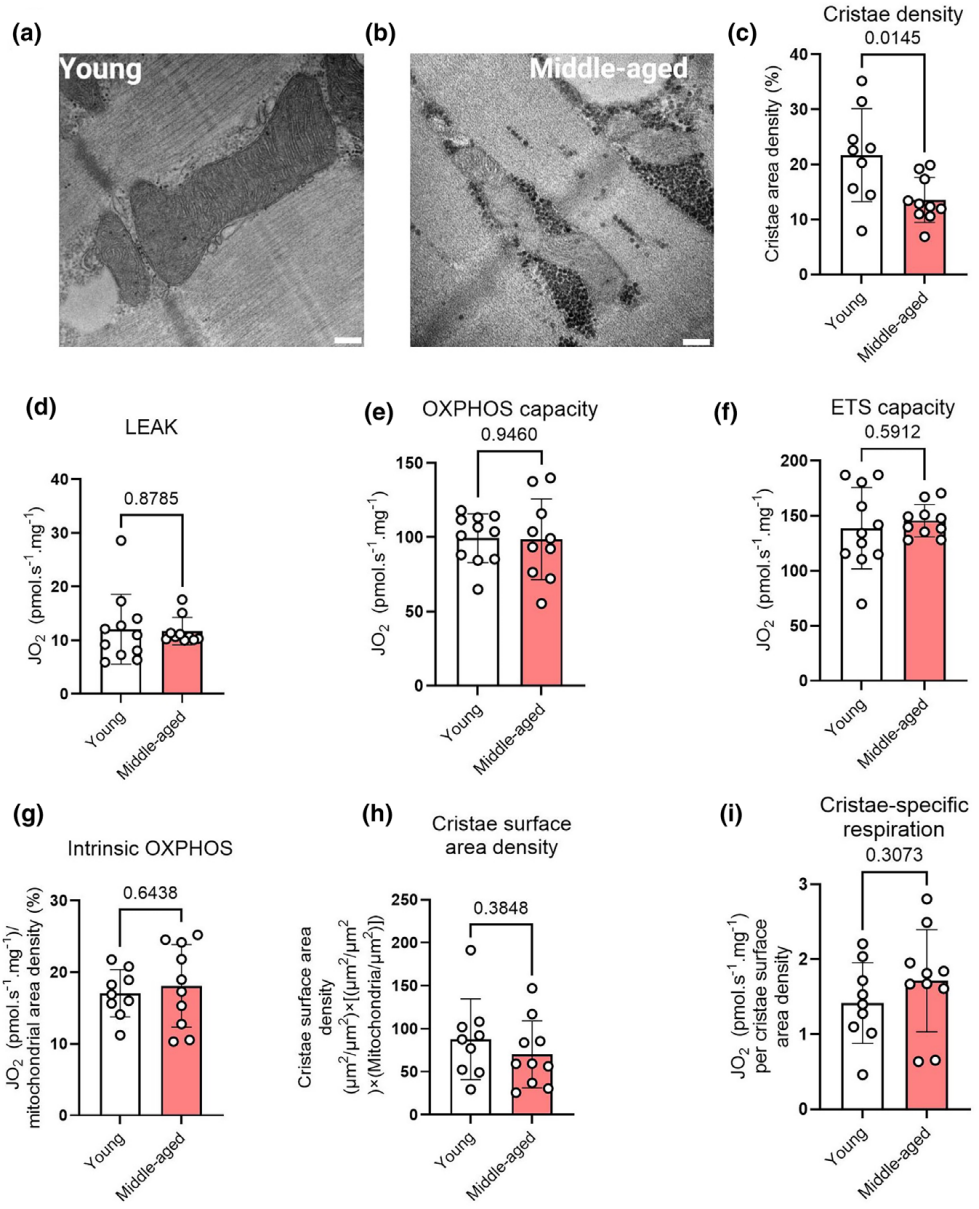
Representative mitochondrial profiles from a young and middle‐aged participant are displayed in (a) and (b), respectively (scale bar = 200 nm). (c), mitochondrial cristae density; (d), leak respiration; (e), oxidative phosphorylation capacity; (f), electron transport system capacity; (g), oxidative phosphorylation capacity normalized by mitochondrial density; (h), cristae surface area density; (i), cristae‐specific oxidative phosphorylation capacity. ETS, electron transport system capacity; JO_2_, oxygen consumption per mg muscle tissue; LEAK, leak respiration; OXPHOS, oxidative phosphorylation capacity. Circles reflect individual data points for each participant. Columns and error bars reflect means ± SD. *p* values displayed above each panel.

The age‐dependent differences in skeletal muscle mitochondrial fragmentation raised the question of whether the mitochondrial cristae ultrastructure was also impacted by age. Skeletal muscle mitochondrial cristae density appears to display plasticity with exercise (Schytz et al., 2024), however, whether ageing affects cristae density in humans is unknown. To this end, we obtained high‐magnification images of individual mitochondrial profiles to quantify mitochondrial cristae density (Figure 3). Mitochondria from young individuals tended to display dense, well‐organized cristae throughout the entire organelle (Figure 3a), whereas the mitochondria from middle‐aged individuals displayed less dense cristae, and regions without cristae (Figure 3b). At the group level, middle‐aged participants displayed a lower mitochondrial cristae density (Figure 3c), evincing age‐associated deterioration at the level of the individual mitochondrion.

Since cristae contain the mitochondrial respiratory enzymes, we next determined the functional implications of the age‐related mitochondrial structural alterations that we observed. Mitochondrial respiration was assessed ex vivo in bundles of permeabilized skeletal muscle fibres using high‐resolution respirometry in hyperoxic conditions to avoid diffusion limitation for oxygen that could contribute to exercise intolerance in vivo, along with saturating substrate concentrations. We assessed mitochondrial respiration in multiple respiratory states, including leak respiration, oxidative phosphorylation capacity and ETS capacity. However, no age‐related differences in mitochondrial respiration were observed across all respiratory states (Figure 3d–f).

A lower cristae density is typically linked to lower respiration per mitochondrion, due to a lower respiratory surface area per mitochondrion. Despite this, oxidative phosphorylation capacity normalized by mitochondrial density did not differ between groups (Figure 3g, Figure S6A–P). However, such methods do not take into account differences in cristae density or mitochondrial number, and it has been shown that when respiration values are normalized by cristae surface area density (i.e., the product of mitochondrial density and cristae density), differences in intrinsic mitochondrial respiration between endurance‐trained and untrained individuals are abolished (Schytz et al., 2024). However, typically employed methods (Schytz et al., 2024) to calculate intrinsic respiration per mitochondrion assume that the overall mitochondrial content is similarly comprised between different populations, whereas the older individuals in the present study were characterized by a greater number of mitochondria per μm^2^, that were, on average, smaller. To account for this, we corrected cristae surface area density by the number of mitochondria per μm^2^ before normalizing oxidative phosphorylation capacity. Our results showed that both corrected mitochondrial cristae surface area density (Figure 3h) and oxidative phosphorylation capacity per cristae surface area (Figure 3i) did not differ between the two groups. Hence, this demonstrates that the lower cristae density per mitochondrion seen in older individuals is mitigated by the overall greater number of intermyofibrillar mitochondria per μm^2^, which collectively explains the lack of differences in both overall and intrinsic mitochondrial respiration between groups. Together, these findings suggest that ageing is associated with ultrastructural alterations within individual mitochondria themselves, but that the effect of these changes on mitochondrial respiration is mitigated by an increased number of intermyofibrillar mitochondria per unit muscle area.

### 2.4 Nutrient accumulation and age‐associated changes in mitochondrial morphology

**FIGURE 4:**
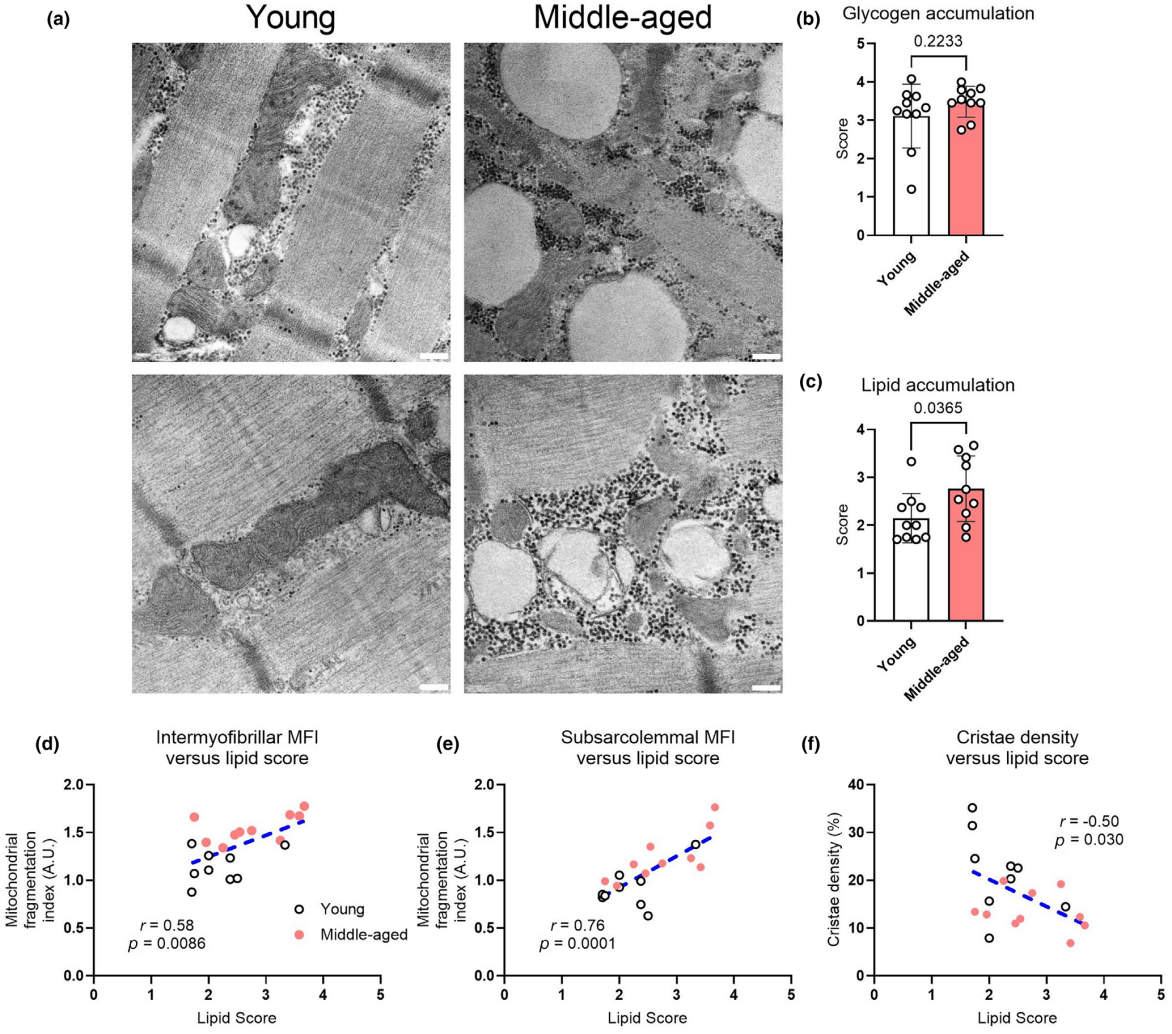
Representative images of nutrient accumulation from young and middle‐aged participants are displayed in (a). (b), glycogen accumulation; (c), lipid accumulation. The lipid score was positively associated with the intermyofibrillar (d) and subsarcolemmal mitochondrial fragmentation indexes (e), whereas lipid score correlated inversely with cristae density (f). *r* and *p* values are displayed on the inset of each panel. Blue lines indicate line of best‐fit.

To investigate the mechanisms underpinning the age‐associated changes in mitochondrial morphology, we assessed intramyocellular skeletal muscle glycogen and lipid droplet accumulation in our electron microscopy images. We reasoned that differences in intramyocellular lipid accumulation might be linked to the smaller and more fragmented mitochondria in the middle‐aged humans (Eggelbusch et al., 2024). Representative images from both groups are shown in Figure 4a. In total, 160 images were scored on a 0–5 scale by three independent blinded assessors. Whilst no between‐group differences were observed with respect to glycogen accumulation (Figure 4b), middle‐aged individuals displayed a greater degree of intramyocellular lipid droplet deposition in skeletal muscle (Figure 4c). Lipid droplet accumulation was positively correlated with fragmentation of mitochondria within both the intermyofibrillar (Figure 4d) and subsarcolemmal regions (Figure 4e), and was negatively associated with mitochondrial cristae density (Figure 4f). No significant correlations were found between glycogen accumulation and indices for mitochondrial structure or function (data not shown). These results highlight an association between age‐related mitochondrial morphological changes and lipid droplet accumulation in middle‐aged humans.

### 2.5 Mitochondrial morphology is associated with an age‐associated reduction in physical capacity

**FIGURE 5:**
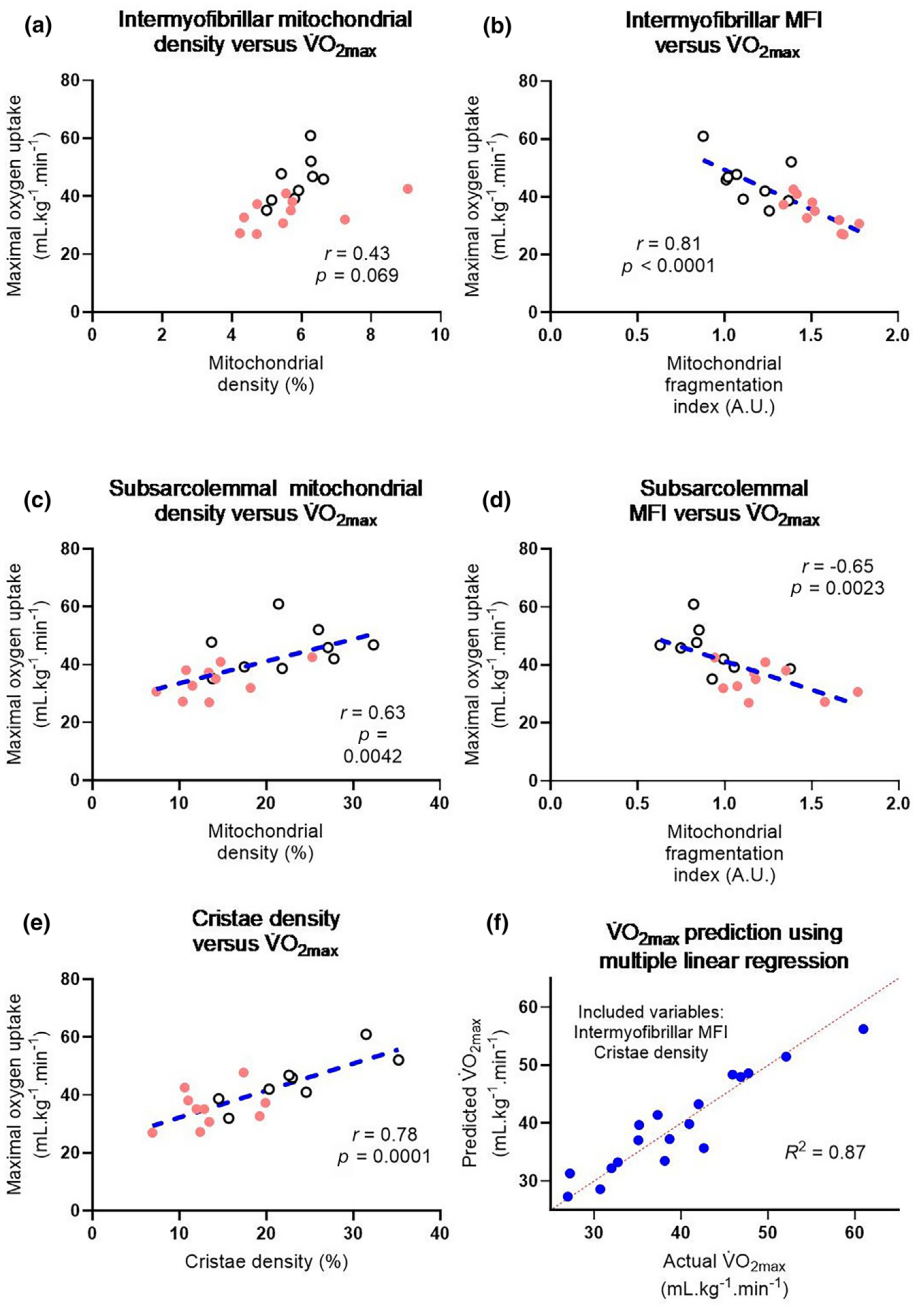
Relationships between maximal oxygen uptake ($\dot{V}$O_2max_) and intermyofibrillar mitochondrial density (a); intermyofibrillar mitochondrial fragmentation index (b); subsarcolemmal mitochondrial density (c); subsarcolemmal mitochondrial fragmentation index (d); and cristae density (e). Open circles represent young individuals, filled circles represent middle‐aged individuals, blue dashed lines represent lines of best‐fit. *r* and *p* values are displayed on the inset of each panel. (f) multiple linear regression analysis performed to understand predictors of $\dot{V}$O_2max_. The intermyofibrillar mitochondrial fragmentation index and cristae density were included in the model. The variance inflation factor score was 2.056. MFI, mitochondrial fragmentation index.

To determine whether mitochondrial characteristics were responsible for the age‐associated physical decline, we performed correlation and multiple linear regression analyses to better understand predictors of $\dot{V}$O_2max_ and O_2_ extraction with age (Figure 5, Figures S7–S10). Whilst intermyofibrillar mitochondrial density was not significantly associated with $\dot{V}$O_2max_ (Figure 5a), the intermyofibrillar mitochondrial fragmentation index negatively correlated with $\dot{V}$O_2max_ (Figure 5b), implying that poorer physical function and exercise capacity was associated with a greater fragmentation of the intermyofibrillar mitochondrial network. In contrast to the intermyofibrillar mitochondrial pool, both the subsarcolemmal mitochondrial density (Figure 5c) and the subsarcolemmal mitochondrial fragmentation index (Figure 5d) were correlated with $\dot{V}$O_2max_. Cristae density was also strongly positively related to $\dot{V}$O_2max_ (Figure 5e). A multiple linear regression model which included only two factors, the intermyofibrillar mitochondrial fragmentation index and mitochondrial cristae density, explained 87% of the variance associated with $\dot{V}$O_2max_ (Figure 5f). Additionally, the subsarcolemmal mitochondrial density was positively related to the slope of muscle O_2_ extraction during exercise (Figure S8C,G), and a multiple linear regression model consisting of capillary density, capillary‐to‐fibre ratio, subsarcolemmal mitochondrial density and cristae density explained 66% of the variance associated with muscle O_2_ extraction during exercise (Figure S8I). These findings are consistent with the theory that the subsarcolemmal mitochondrial population provides the proton motive force via O_2_ consumption (Parry et al., 2024), and that the intermyofibrillar mitochondrial population provides the ATP generation by ATP synthase where energetic demand is the highest during contractions (Glancy et al., 2015). Hence, our observation of age‐related intermyofibrillar mitochondrial fragmentation and lower subsarcolemmal mitochondrial density are suggestive of an impaired cellular energy distribution between mitochondrial populations within the mitochondrial reticulum (Glancy et al., 2015), which could in turn impair the maximal rate of oxidative ATP generation and muscle O_2_ extraction during exercise, respectively. Finally, subsarcolemmal mitochondrial density was strongly negatively associated with chronological age, whereas the intermyofibrillar mitochondrial fragmentation index displayed a strong positive correlation with chronological age (Figure S9). Taken together, our findings show that mitochondrial alterations in skeletal muscle can potentially explain a large proportion of the age‐associated loss of physical capacity, and these age‐associated mitochondrial changes progressively worsen with advancing age.

## 3 DISCUSSION

In the present study, we determined the relationships between skeletal muscle mitochondrial characteristics and physical decline by comparing young and middle‐aged individuals. We identified that, although intermyofibrillar mitochondrial density did not differ between groups, early ageing was associated with a more fragmented mitochondrial network, a lower subsarcolemmal mitochondrial density and a reduced mitochondrial cristae density. Furthermore, fragmentation of the intermyofibrillar mitochondrial network, subsarcolemmal mitochondrial density and mitochondrial cristae density were the strongest predictors of markers of physical capacity across the middle‐age range. These findings highlight that mitochondrial phenotypes representative of early ageing, such as fragmentation, loss of cristae density and loss of subsarcolemmal mitochondrial density, can precede the development of overt skeletal muscle mitochondrial dysfunction which can occur at advanced ages. Collectively, therefore, these findings highlight the central role of skeletal muscle mitochondrial alterations in age‐associated physical decline.

### 3.1 Age‐related changes in skeletal muscle mitochondria

Numerous preclinical models have demonstrated that mitochondrial function and morphology are critical mediators of the ageing process and muscle mass (Romanello et al., 2010), and interventions that enhance mitochondrial function have been shown to mitigate age‐related physiological decline (Mills et al., 2016). In line with this work in non‐human models, our data support the notion that mitochondrial health is a central determinant of the skeletal muscle ageing process and the age‐related decline in physical function. Despite this extensive preclinical evidence, however, the human data are more controversial. For instance, whilst studies have demonstrated declines in mitochondrial function with age (Gonzalez‐Freire et al., 2018), conflicting reports abound, with many studies showing no differences in mitochondrial respiration and enzyme activity between young and older individuals (Distefano et al., 2017). Moreover, human data linking mitochondrial deterioration with age‐related physical decline is sparse, and almost solely limited to respiratory measurements (Gonzalez‐Freire et al., 2018), which can conceal age‐related mitochondrial morphological alterations that develop before overt mitochondrial respiratory dysfunction. For example, fragmentation of mitochondrial networks in skeletal muscle causes insulin resistance (Jheng et al., 2012), increases muscle protein degradation and loss of muscle mass (Romanello et al., 2010), and occurs after 6 days of bed rest in humans, prior to the loss of mitochondrial content and oxidative capacity that occurs after long‐term bed rest (Eggelbusch et al., 2024). Indeed, our data confirm this, as intermyofibrillar mitochondrial density was not different between groups, whereas the older participants displayed more fragmented intermyofibrillar mitochondria, consisting of more abundant but smaller mitochondria, alongside a reduction in cristae density. These intrinsic alterations in mitochondrial morphology likely occur prior to the reduction in intermyofibrillar mitochondrial density and respiratory capacity known to occur in later years (Conley et al., 2000; Short et al., 2005).

### 3.2 Reduced maximal oxygen uptake with ageing

Consistent with others, we found a lower muscle O_2_ extraction and $\dot{V}$O_2max_ in middle‐aged individuals (McGuire et al., 2001). Muscle O_2_ extraction is determined by the ratio of blood flow ($\dot{Q}$) to $\dot{V}$O_2_, however, given the evidence of impaired maximal $\dot{Q}$ (Poole et al., 2003), submaximal $\dot{Q}$ (Proctor et al., 1998) and $\dot{Q}$ maldistribution with ageing (Musch et al., 2004), a greater $\dot{Q}$/$\dot{V}$O_2_ is unlikely to explain blunted muscle O_2_ extraction with ageing. Instead, this finding is likely explained by a reduced capacity for diffusive O_2_ supply (DmO_2_). Our data show an unaltered structural capacity for O_2_ diffusion assessed via capillarization, which is consistent with some reports in the human literature (Chilibeck et al., 1997) and the observation in animals that capillarization is maintained in excess of fibre oxidative capacity with ageing (Mathieu‐Costello et al., 2005). We also found an increased total [heme] amplitude from rest to maximal exercise in middle‐aged subjects, owing to reduced baseline total [heme]. This finding is consistent with reports of altered capillary hemodynamics in aged rat muscle (Copp et al., 2009), which have been suggested to impair blood‐myocyte diffusive O_2_ flux (Poole et al., 2006). However, measurement of the absolute concentrations of heme chromophores during exercise with ageing using time‐resolved NIRS (Koga et al., 2015) should be performed in order to clarify these findings. Importantly, however, exercise‐induced changes in DmO_2_ supporting elevated O_2_ flux are only partly explained by events occurring in the capillary (Hirai et al., 2017). Instead, we propose that the intracellular alterations observed herein, specifically reduced subsarcolemmal mitochondrial density and intermyofibrillar mitochondrial fragmentation, may be partly implicated in impairments in capillary‐myocyte diffusional O_2_ flux with ageing.

### 3.3 Associations between mitochondrial characteristics and physical function

Among the strongest links between mitochondria‐associated indices and functional physiological outcome variables was the positive relationship between subsarcolemmal mitochondrial density and muscle O_2_ extraction, and the inverse association between the intermyofibrillar mitochondrial fragmentation index and $\dot{V}$O_2max_. The subsarcolemmal mitochondria are physically located close to capillaries where O_2_ is delivered, and extend in reticulum‐like fashion towards the core of the myofibres (Glancy et al., 2015, 2017). This mitochondrial population is thought to be critically involved in generation of the proton motive force in regions located in close proximity to the capillaries, which is then transmitted towards the core of the fibre where the energetic demand is greatest (Glancy et al., 2015, 2017). This mechanism is thought to help mitigate the importance of O_2_ diffusion distances in skeletal muscle, with propagation of the mitochondrial membrane potential in response to changes in energetic demand helping to prevent the formation of anoxic loci. We did not find any evidence of altered capillarization between groups, hence the altered O_2_ extraction between groups must have been related to either alterations in capillary hemodynamics (Copp et al., 2009), intramuscular $\dot{Q}$/$\dot{V}$O_2_ heterogeneity (Musch et al., 2004) or intracellular changes. Of note, our observation of a lower subsarcolemmal mitochondrial density in middle‐aged individuals was positively related to muscle O_2_ extraction across groups. This finding is consistent with an effect of ageing on intracellular DmO_2_, and the distinct bioenergetic role that this mitochondrial subpopulation plays in energy production (Parry et al., 2024). However, such notions remain speculative at present, and further research is clearly necessary to elucidate the physiological role of mitochondrial subpopulations in vivo.

The fragmentation of the intermyofibrillar mitochondria would also likely serve to undercut physical capacity. For instance, mitochondrial network connectivity enables rapid intracellular mitochondrial communication and distribution of the proton motive force throughout the cell (Glancy et al., 2017). Hence, whilst in the healthy state these networks can facilitate rapid adjustments in ATP supply to fuel contractile demand, fragmentation of this network can compromise cellular energy conversion (Glancy et al., 2017). Due to mitochondrial fragmentation a disturbed distribution of the mitochondrial membrane potential in response to alterations in muscle ATP demand would be expected to exacerbate the degree of intracellular perturbation (i.e., greater Δ[ADP], Δ[inorganic phosphate], Δ[phosphocreatine]) required to sustain a given $\dot{V}$O_2_, which could explain the exacerbated fatigability of elderly muscle. Our data therefore suggest that mitochondrial fragmentation may be an early sign of impaired mitochondrial energetics in vivo. Collectively, we propose that ageing results in a loss of subsarcolemmal mitochondrial density and intermyofibrillar mitochondrial fragmentation prior to an overt loss of muscle oxidative capacity, but that both of these alterations hinder physical capacity via effects on intracellular energetic communication during muscular activity.

### 3.4 Ageing and skeletal muscle mitochondrial cristae density

Our electron microscopy data regarding mitochondrial cristae density are consistent with the extant human gene expression (Kurochkina et al., 2024), proteomics (Ubaida‐Mohien et al., 2022) and animal data (Simon et al., 1969) documenting age‐related changes in cristae morphology and their important role in shaping muscle function. Ubaida‐Mohien et al. (2022) demonstrated that proteins regulating the mitochondrial contact site and cristae organizing system (MICOS) complex were over‐represented in the muscle of high‐functioning octogenarians. Moreover, recent transcriptomic and proteomic results that distinguished between genes and proteins associated with physical inactivity and inflammation versus those associated with primary ageing (Kurochkina et al., 2024) showed that most of the differentially expressed genes and proteins between young and old individuals could be attributed to physical inactivity and/or chronic inflammation, whereas those associated with primary ageing included mitochondrial respiratory enzymes, mitochondrial complex assembly factors, regulators of cristae formation and mitochondrial reactive oxygen species production (Kurochkina et al., 2024). Consistent with altered expression of genes regulating cristae formation in humans (Kurochkina et al., 2024), alterations in cristae morphology have been observed in aged mouse cardiac muscle (Vue et al., 2023) and flight muscle of houseflies (Simon et al., 1969), but not yet in humans. Moreover, overexpression of optic atrophy factor‐1 (OPA1, a regulator of cristae remodelling) has been shown to ameliorate age‐related damage in multiple organs (Varanita et al., 2015). Hence, this work demonstrates that the effects of human ageing extend to the individual mitochondrion, and that cristae remodelling is strongly related to age‐related alterations in physical capacity.

### 3.5 Effects of primary ageing versus physical activity

In this study we chose to focus on active middle‐aged individuals, as we reasoned that detecting mitochondrial changes early in the ageing process, before the development of substantial age‐related atrophy or physical impairment, would help dissect differences between the effects of early and late ageing on skeletal muscle metabolism, function and mitochondrial health. In this regard, our middle‐aged group did not display the typical reductions in muscle fibre cross‐sectional area associated with the later stages of ageing (Tieland et al., 2018), and all participants were physically active and functional. Comparison with published reference values for $\dot{V}$O_2max_ indicated that the middle‐aged group in the present study were in the 50–75th (males) and 90–95th (females) percentiles for $\dot{V}$O_2max_, versus the 25–50th and 50–75th percentiles for males and females in the young group, respectively (Kaminsky et al., 2015). This suggests that the age‐related mitochondrial fragmentation observed herein appears not to be related to the reduced physical activity that so often occurs with ageing. The corollary of this interpretation is that maintaining a recreational level of physical activity is not sufficient to offset the effects of ageing on skeletal muscle mitochondrial health and physical function. Indeed, this suggestion is consistent with the strong positive relationships between mitochondrial fragmentation and age (Figure S9), implying that the muscle mitochondrial pool becomes progressively more fragmented with advancing age. Hence, it is likely that our findings reflect an early ageing phenotype, making the mitochondrial changes observed herein strong candidates for intervention studies aiming to slow the progression of the effects of ageing on physical function. What is not known, however, is the degree to which exercise can offset such mitochondrial changes. McGuire et al., 2001 showed that 6 months of endurance training was sufficient to reverse the effects of 30 years of ageing on $\dot{V}$O_2max_, an effect largely attributable to increased maximal O_2_ extraction. Considering the effects of acute exercise on mitochondrial fusion (Huertas et al., 2019), whether improvements in $\dot{V}$O_2max_ with training in older subjects can be explained by a reversal of mitochondrial fragmentation would be an interesting line of further research.

### 3.6 Limitations

This study is cross‐sectional in nature, and therefore cannot establish the causality of ageing per se. For logistical reasons a different measure of physical activity was employed in some participants. However, when comparing young versus middle‐aged participants with either measure, there were no differences in physical activity levels (i.e., see Table S1). Moreover, that the middle‐aged participants occupied a higher $\dot{V}$O_2max_ percentile with respect to their peers of a similar age than the younger participants suggests that the middle‐aged participants were somewhat more active than the younger group. The present analyses represent a hybrid of paravascular and subsarcolemmal mitochondrial subpopulations (Parry et al., 2024), which raises the possibility that exercise capacity and subsarcolemmal mitochondrial density would have been more strongly related were the paravascular mitochondria also taken into account. Future research should therefore explore the relationships between paravascular mitochondrial density and exercise capacity. Although we speculate a bioenergetic impact of the structural mitochondrial changes that we observed, we did not demonstrate any differences in mitochondrial respiration between groups. We suggest that the lack of group differences observed here reflects that there were no differences in intermyofibrillar mitochondrial density, as subsarcolemmal mitochondria occupy a much smaller fraction of total cell volume than intermyofibrillar mitochondria. Moreover, we assessed maximal respiration at saturating [ADP], which is several‐fold higher than that typically observed in vivo. Hence, our data are still consistent with an effect of the morphological and ultrastructural mitochondrial changes documented herein on physical capacity in vivo, and this is supported by the strong relationships reported between these variables. Finally, our respiration data were assessed at supra‐physiological PO_2_, and future research could therefore utilize ^31^phosphorous‐magnetic resonance spectroscopy or intermittent arterial occlusions using NIRS to delineate the impact of mitochondrial fragmentation on energetic function in vivo.

## 4 CONCLUSIONS

In conclusion, our study advances our understanding of the contribution of skeletal muscle mitochondrial alterations to early biological ageing by demonstrating strong relationships between mitochondrial characteristics and whole body physical capacity. Specifically, ageing was associated with a lower $\dot{V}$O_2max_ and reduced capacity for muscle O_2_ extraction, and this reduced physical capacity occurred alongside a fragmentation of the intermyofibrillar mitochondrial network and a lower subsarcolemmal mitochondrial density. Older participants displayed a lower cristae density per mitochondrion, which was partially compensated for by more intermyofibrillar mitochondria per area. Collectively, the extent of mitochondrial fragmentation, cristae density and subsarcolemmal mitochondrial density explained a majority of the variance in physical capacity across age groups. Taken together, our work buttresses preclinical studies indicating an important role of mitochondria in skeletal muscle ageing. Novel interventions that target mitochondrial health are a promising avenue for mitigating and reversing skeletal muscle ageing.

## 5 METHODS

The *Extended Methods* section in the supplemental file describes the methods of this study in more detail.

### 5.1 Participants

Twelve young and ten middle‐aged participants volunteered to take part. The experiment was approved by the Medical Ethics Committee of Vrije Universiteit Medical Center (VUmc) and the Medical Ethics Committee of the Amsterdam UMC, and conformed to the Declaration of Helsinki. The study was registered with the Netherlands National Trial Register (trial number: NL9583) and www.clinicaltrials.gov (NCT05225688) (Appelman et al., 2024), respectively.

### 5.2 Experimental overview

Each participant visited the laboratory on two separate occasions. At the initial visit, participants completed an incremental ramp exercise test on a cycle ergometer (Lode Excalibur Sport, Lode, Groningen, The Netherlands) for determination of $\dot{V}$O_2max_, exercise capacity and other variables related to in vivo aerobic function. For the second visit, a vastus lateralis muscle biopsy was taken. Muscle biopsies were taken from the vastus lateralis at one‐third of the distal length using a suction‐supported Bergström needle. Biopsy samples were immediately frozen in liquid nitrogen until subsequent analysis or stored as otherwise stated. Prior to the first visit, participants enrolled in trial NL9583 (*n* = 12) completed a 7 day physical activity questionnaire, whereas participants enrolled in trial NCT05225688 (*n* = 10) wore an accelerometer (Actigraph wGT3X‐BT, 60 Hz sampling frequency) on the right hip over a 14‐day period, as described previously (Appelman et al., 2024).

#### 5.2.1 Exercise testing procedures

The test began with 2 min of rest on the ergometer followed by a 4 min baseline cycling period at 30 (females) or 40 (males) W, followed by a ramped, linear increase in power output at a rate of 20–30 W.min^−1^ (depending on sex, body mass and anticipated fitness) until the limit of tolerance. Participants were instructed to maintain their cadence between 70 and 90 revolutions/min, and task failure was defined as the point at which cadence dropped <60 revolutions/min despite verbal encouragement. Pulmonary gas exchange and ventilation were measured on a breath‐by‐breath basis throughout the entire test (Cosmed Quark CPET; Cosmed, Rome, Italy). Muscle deoxygenation was noninvasively recorded throughout the test via the deoxygenated [heme] (deoxy[heme]) signal obtained from continuous‐wave near‐infrared spectroscopy (NIRS, Portamon, Artinis, The Netherlands). For further details on analysis procedures from this test, see the *Extended Methods*.

#### 5.2.2 Near‐infrared spectroscopy

Participants wore a spatially‐resolved, continuous‐wavelength NIRS device (PortaMon, Artinis Medical Systems, The Netherlands) midway along the belly of the vastus lateralis muscle. The device measures relative changes in the concentrations of total‐ and deoxy[heme], and a physiological calibration involving an occlusion procedure was performed in order to facilitate between‐group comparisons. All values during the exercise test were expressed as a fraction of the difference between the maximal deoxygenation value during the occlusion and the minimum value following reperfusion. Individual Δdeoxy[heme] data were determined as a function of absolute and relative power output. For further details on the analysis procedures of the NIRS data, see the extended methods.

#### 5.2.3 Succinate dehydrogenase activity

Histochemistry was carried out on 10 μm sections cut from the biopsies. SDH activity was determined using quantitative histochemistry. Directly after sectioning, slides were air‐dried for 10 min, and incubated at 37°C in a medium containing 0.55 mM tetranitroblue tetrazolium (Sigma, St. Louis, USA), 0.2 M sodium succinate, 14 mM sodium azide and 0.1 M sodium phosphate buffer, pH 7.6 for 20 min in the dark. The reaction was briefly stopped in 0.01 M HCl, before sections were washed and mounted with glycerine gelatin. Samples were stored at 4°C and images were made within 10 days of staining using a light microscope (Leica DMRB, Wetzlar, Germany) using calibrated grey filters and an individual calibration curve at 660 nm. The averaged SDH activity was obtained by outlining individual skeletal muscle fibres and absorbance was assessed using ImageJ (NIH, Bethesda, USA). Values were expressed as ΔA_660_ per μm tissue thickness per second of staining time (ΔA_660_·μm^−1^·s^−1^).

#### 5.2.4 Capillarization

Capillarization was assessed by staining for *Ulex Europaeus* Agglutin 1 lectin (UEA‐1). Sections were air‐dried for 10 min and then fixed in ice cold acetone (−20°C) for 15 min. Subsequently, slides were washed three times for 2 min in 1x PBS and blocked with 0.1% bovine serum albumin (SP‐505 Vector Laboratories, Inc., Burlingame, CA, USA) for 30 min. Afterwards, slides were incubated with 20 μg/mL UEA‐I (B‐1065‐2, Vector Laboratories) for 30 min at room temperature. Following this another washing step was performed, followed by incubation with VECTASTAIN® Elite ABC‐HRP Kit Peroxidase (PK‐6100, Vector Laboratories) for 30 min at room temperature. Another washing step was performed, followed by incubation with ImmPACT™ AMEC Red Peroxidase Substrate (SK‐4285, Vector Laboratories) for 10 min at room temperature. Finally, a washing step was performed in ddH_2_O for 5 min and sections were mounted with glycerine‐gelatin (pre‐warmed to 37°C). Capillary density (the number of capillaries per mm^2^) and the capillary‐to‐fibre ratio were calculated using ImageJ.

#### 5.2.5 Transmission electron microscopy

Samples were fixed in 2.5% glutaraldehyde and before embedding were washed, fixated in 1% osmium tetroxide and 1.5% potassium ferrocyanide for 1 h, washed, and dehydrated with ethanol (i.e., 2 × 70, 80, 90% and 2% × 100% ethanol for 15 min each). Samples were impregnated in propylene oxide/Epon812 (1:1) for 1 h, followed by Epon812 at 37°C for 30 min. Finally, samples were embedded longitudinally in moulds containing fresh Epon812 and left to polymerize for 3 days at 65°C. Ultra‐thin (10 nm) longitudinal sections were cut, collected on 150‐mesh copper grids, and stained with 3.5% (w/v) uranyl acetate (5 min) and 3% (w/v) lead citrate (5 min). Visualization was performed with a FEI Tecnai 120 kV Transmission electron microscope (TEM, ThermoFisher Scientific, Waltman, Massachusetts, VS) using a Veleta camera Plus. Four individual fibres were imaged per subject, with four images of the subsarcolemmal region and four images of the intermyofibrillar region being taken per fibre. Images were taken in a randomized, systematic fashion, at a magnification of 18,500× for quantification of mitochondrial area density and morphology, and at 48,500× for cristae density measurements and analysed with ImageJ. Further details on the analysis of the mitochondrial variables derived from the TEM analysis may be found in the *Extended Methods*. Mitochondrial cristae ultrastructure was determined by manually tracing the outlines of cristae in eight randomly selected mitochondrial profiles in ImageJ. The images used for the cristae analysis were also scored on a scale from 0 (no lipid, little glycogen) to 5 (extreme lipid and glycogen accumulation) for glycogen and intramyocellular lipid deposition by three independent raters in a blinded fashion, as previously described (Eggelbusch et al., 2024).

#### 5.2.6 High‐resolution respirometry

Mitochondrial respiration was assessed in permeabilized skeletal muscle fibres (for full protocol see *Extended Methods*). Freshly isolated fibres were permeabilized with saponin (50 μg.mL^−1^) for 20 min at 4°C in a solution containing (in mM) CaK_2_EGTA (2.7), K_2_EGTA (7.23), Na_2_ATP (5.77), MgCl_2_·6H_2_O (6.56), taurine (20), Na_2_Phosphocreatine (15), imidazole (20), dithiothreitol (0.5) and MES (50) (pH 7.1). Fibres were then washed in respiration solution containing: EGTA (0.5), MgCl_2_·6H_2_O (3.0), K‐lactobionate (60), taurine (20), KH_2_PO_4_ (10), HEPES (20), sucrose (110) and 1 g.L^−1^ BSA (pH 7.1). Fibres were then blotted dry, weighed and transferred to a respirometer (Oxygraph‐2 k, Oroboros, Innsbruck, Austria) in respiration medium at 37°C. Oxygen concentration was between 300 and 500 μM throughout all experiments to avoid limitations in oxygen supply (i.e., an approximate PO_2_ of 175–300 mmHg). Background respiration was assessed before adding substrates and was subtracted from all subsequent values. Leak respiration was assessed after the addition of sodium glutamate (10 mM), sodium malate (0.5 mM) and sodium pyruvate (5 mM). NADH‐linked respiration was assessed after the addition of 5 mM ADP and 10 mM cytochrome c to account for outer mitochondrial membrane integrity. Maximal oxidative phosphorylation (OXPHOS) capacity was assessed after the addition of 10 mM succinate. ETS capacity was determined via titration of carbonylcyanide‐4‐trifluoro‐ methoxyphenylhydrazone (FCCP) in 0.5 μM steps until no further increase in oxygen consumption was observed. Succinate‐linked respiration (S + ROT) was measured following the addition of 0.5 μM rotenone. Respiration experiments were performed in duplicate or triplicate depending on tissue availability, and the results were averaged. Respiration values were normalized to wet weight and expressed in pmol O_2_.s^−1^.mg^−1^. Three separate approaches were employed to assess intrinsic mitochondrial respiration (i.e., mitochondrial quality), independent of mitochondrial abundance: (1) normalization to the unique intermyofibrillar mitochondrial area density from the TEM analysis; (2) via calculation of respiratory control ratios; and (3) by normalizing by the cristae surface area density in order to yield cristae specific respiration. Further details on these procedures are provided in the *Extended Methods*.

### 5.3 Statistical analyses

All data are presented as group means ± SD unless otherwise indicated. Data normality was tested using a Shapiro‐Wilkes normality test. Differences between group means were assessed using independent samples *t* tests when data were normally distributed and Mann–Whitney *U* test when non‐normally distributed. Correlations between variables were assessed using Pearson's correlation coefficient. Multiple linear regressions were used for variables hypothesized to influence $\dot{V}$O_2max_ and muscle O_2_ extraction. Any variable with a Variance Inflation Factor score >5 was subsequently excluded from the model due to multicolinearity. Density distributions were obtained distribution using R studio with the ‘geom_density’ function of ggplot2 with a Gaussian kernel for density estimation. All other statistical analysis was performed using GraphPad Prism version 9 (GraphPad Software, La Jolla, CA). Statistical significance was accepted when *p* < 0.05.

## AUTHOR CONTRIBUTIONS

RPG and RCIW conceived the research. The research was designed by RPG, RCIW, BA, MvV and BC. RPG, BC, EB, MvdL, AS, AK, BA, AEG, NNvdW, JJdK and FWB conducted the experiments. RPG, BC and EB analysed the results. RPG drafted the manuscript, and the figures were prepared by RPG and RCIW. All authors interpreted the data, critically revised the manuscript, and approved the final version for submission.

## FUNDING INFORMATION

This work was supported by the European Foundation for the Study of Diabetes (EFSD/Boehringer Ingelheim European Research Programme on “Multi‐System Challenges in Diabetes” 2020, RCIW and RPG) and Amsterdam Movement Sciences (2023, RPG).

## CONFLICT OF INTEREST STATEMENT

None declared.

## Supporting information


Appendix S1.

## Data Availability

Further data supporting the conclusions drawn in this study may be found in the supplementary material. Additional data that support the findings of this study are available on request from the corresponding authors.
