# Supplementary material for: Skeletal muscle mitochondrial fragmentation predicts age‐associated decline in physical capacity
Source: Aging Cell. 2024 Dec 4;24(2):e14386. doi: 10.1111/acel.14386 (PMC11822651; doi:10.1111/acel.14386)
Supplement: Supplementary file 1 — Appendix S1. [file ACEL-24-e14386-s001.docx]

**Supplementary file related to:**

**SKELETAL MUSCLE MITOCHONDRIAL FRAGMENTATION PREDICTS AGE-ASSOCIATED DECLINE IN PHYSICAL CAPACITY**

Richie P. Goulding^1^**,** Braeden T. Charlton^1^, Ellen A. Breedveld^1^, Matthijs van der Laan^1^, Anne R. Strating^1^, Wendy Noort^1^, Aryna Kolodyazhna^1^, Brent Appelman^2,3^; Michèle van Vugt^2,4^, Anita E. Grootemaat^5^, Nicole N. van der Wel^5^, Jos J. de Koning^1^, Frank W. Bloemers^6^, Rob C.I. Wüst^1^

^1^Department of Human Movement Sciences, Faculty of Behavioral and Movement Sciences, Vrije Universiteit Amsterdam, Amsterdam Movement Sciences, Amsterdam, Netherlands

^2^Center for Experimental and Molecular Medicine, Amsterdam UMC location University of Amsterdam, Amsterdam, Netherlands;

^3^Amsterdam Institute for Infection and Immunity, Amsterdam, the Netherlands;

^4^Division of Infectious Diseases, Tropical Medicine, Department of Medicine, Amsterdam UMC location University of Amsterdam, Amsterdam, the Netherlands;

^5^Electron Microscopy Centre Amsterdam, Amsterdam UMC, Location Academic Medical Centre, Amsterdam, Netherlands;

^6^Department of Trauma Surgery, Amsterdam Movement Sciences, Amsterdam UMC, Vrije Universiteit Amsterdam, Amsterdam, Netherlands.

**Running title**: Skeletal muscle mitochondrial fragmentation and ageing

**Corresponding authors:** Richie Goulding and Rob CI Wüst, Department of Human Movement Sciences, Faculty of Behavioural and Movement Sciences, Vrije Universiteit Amsterdam, van der Boechorststraat 7, 1081 BT Amsterdam, The Netherlands. E-mail address: r.p.goulding@vu.nl, r.wust@vu.nl.

**Extended methods**

*Participants*

Twelve young and ten middle-aged participants volunteered to take part. The experiment was approved by the Medical Ethics Committee of Vrije Universiteit Medical Center (VUmc) and the Medical Ethics Committee of the Amsterdam UMC, and conformed to the Declaration of Helsinki. The study was registered with the Netherlands National Trial Register (trial number: NL9583) and www.clinicaltrials.gov (NCT05225688)^1^, respectively.

*Experimental Overview.*

Participants were instructed to avoid alcohol and strenuous exercise 24 h prior to each visit, not to consume caffeine on the same day as a scheduled laboratory visit and to arrive at least 3 h postprandial. Each test was scheduled at the same time of day ± 2 h and laboratory visits were separated by at least 48 h. Each participant visited the laboratory on two separate occasions. At the initial visit, participants completed an incremental ramp exercise test on a cycle ergometer (Lode Excalibur Sport, Lode, Groningen, The Netherlands) for determination of $\dot{V}$O_2max_, exercise capacity, and other variables related to *in vivo* aerobic function. For the second visit, a vastus lateralis muscle biopsy was taken. Prior to the first visit, participants enrolled in trial NL9583 (n=12) completed a 7 day physical activity questionnaire, whereas participants enrolled in trial NCT05225688 (n=10) wore an accelerometer (Actigraph wGT3X-BT, 60 Hz sampling frequency) on the right hip over a 14-day period, as described previously^1^.

*Exercise testing procedures*.

All exercise tests were performed in the same temperature controlled laboratory (18-21 °C and 50 ± 10 % humidity). After the assessment of anthropometric characteristics and instrumentation, the measurements began with a 2 minute period of quiet rest with the participant seated on the ergometer. The test began with a 4 minute period of baseline cycling at 30 (females) or 40 (males) W, followed by a ramped, linear increase in power output at a rate of 20-30 W.min^-1^ until the limit of tolerance was reached. The individual ramp rates were selected based upon the subject’s anthropometric characteristics and self-reported levels of physical activity, with the aim to reach an exercise duration of 8-12 minutes prior to task failure. Participants were instructed to maintain their cadence between 70-90 revolutions/minute, and task failure was defined as the point at which cadence dropped <60 revolutions/minute despite strong verbal encouragement. Pulmonary gas exchange and ventilation were measured on a breath-by-breath basis throughout the entire test using open-circuit spirometry (Cosmed Quark CPET; Cosmed, Rome, Italy). Muscle deoxygenation was noninvasively recorded throughout the test via the deoxygenated [heme] (deoxy[heme]) signal obtained from continuous-wave near-infrared spectroscopy (NIRS, Portamon, Artinis, The Netherlands). Before each test, the gas analyser and volume transducer were calibrated using gases of known concentrations and a 3-litre syringe and according to the manufacturer’s instructions, respectively. Peak power output was determined as the highest power output attained during the test.$\dot{V}$O_2max_ was determined as the highest 30 second rolling average value recorded during the test. Although this $\dot{V}$O_2max_ value was not verified with an additional verification trial^2^, 59% of our participants evinced a plateau in the $\dot{V}$O_2_ response to exercise towards the end of the incremental test, and $\dot{V}$O_2max_ (plateau: 41 ± 9 mL.kg^-1^.min^-1^, no plateau: 41 ± 11 mL.kg^-1^.min^-1^, *P* = 0.93) as well as the percentage of $\dot{V}$O_2max_ at which the GET (plateau: 72 ± 6%, no plateau: 70 ± 7%, *P* = 0.60) or RCP (plateau: 91 ± 8%, no plateau: 87 ± 6%, *P* = 0.25) occurred did not differ between participants that did and did not evince a $\dot{V}$O_2_ plateau. The gas exchange threshold (GET) was determined as an increase in$\dot{V}$CO_2_ with respect to$\dot{V}$O_2_, an increase in the ventilatory equivalent for $\dot{V}$O_2_ without a corresponding increase in the ventilatory equivalent for$\dot{V}$CO_2_, and an increase in the end-tidal O_2_ pressure without a corresponding increase in end-tidal CO_2_ pressure, with correction for the mean response time of $\dot{V}$O_2_ kinetics, as previously described^3^.

*Near-infrared spectroscopy*. The NIRS device employed in the present study (PortaMon, Artinis Medical Systems, The Netherlands) has been described in detail previously^4^. Briefly, the device measures relative changes in the concentrations of total- and deoxy[heme] via continuous-wavelength, spatially-resolved spectroscopy. Data were recorded at 1 Hz using wavelengths of 758 nm and 847 nm with light source-detector separation distances of 30, 35, and 40 mm. The device was placed midway along the belly of the vastus lateralis muscle and fixed in place with tape and bandages to avoid motion artefact and intrusion of extraneous light. To facilitate comparisons between groups, a physiological calibration procedure was performed whereby a cuff was inflated around the inguinal crease of the thigh to suprasystolic pressures (≥300 mmHg). The total [heme] signal was monitored to ensure that there was no arterial inflow, and the occlusion was performed until a clear plateau in the deoxy[heme] signal was evident for at least 30 seconds. Subsequently, the cuff was released and the reperfusion phase was monitored for at least two minutes, or until a plateau occurred. Subsequently, all values during the exercise test were expressed as a fraction of the difference between the maximal value of deoxygenation obtained during the occlusion and the minimum value following reperfusion. Individual Δdeoxy[heme] data were first plotted as a function of time, before conversion to power output. The profiles of Δdeoxy[heme] with respect to absolute (i.e., W) and relative (i.e., percentage of peak power output) power output were then analyzed using a double-linear model, as performed previously^5^. Data used in this analysis included all points between the onset of the systematic rise in Δdeoxy[heme] near the beginning of the test and the end of the test. Piecewise linear regression analysis was then applied and yielded two linear functions:

$$y=m_{1}x+ c_{1}, for x<BP$$

$$y=m_{2}x+ c_{2}, for x>BP$$

Where *m* represents the slope, *c* represents the intercept, and BP represents the intersection of the two lines, determined via the lowest sum of squared errors. The slope of the first linear function thus describes the relative reliance on muscle O_2_ extraction for a given change in power output during the incremental test.

*Muscle biopsy procedures*. Muscle biopsies were taken from the vastus lateralis at approximately one-third of the distal length by an experienced surgeon using a modified Bergström needle technique with suction. The biopsy site was locally anaesthetized using 2% lidocaine solution, and a ~1 cm incision was made through the skin. Biopsy samples were carefully removed and partitioned into three pieces. One ~30 mg piece was aligned according to the fibre arrangement under a light microscope and frozen in liquid nitrogen for histochemistry, another ~5 mg piece was placed in fixative solution for later TEM analysis, and another ~15 mg piece was utilized for high-resolution respirometry experiments. For histochemistry, frozen muscle samples were cut into 10 µm thick sections in transverse orientation in a cryostat at -20^o^C, before being mounted on polylysine-coated slides.

*Succinate dehydrogenase activity.* SDH activity was determined using quantitative histochemistry. Directly after sectioning, slides were air-dried for ten minutes, and incubated at 37^o^C in a medium containing 0.55 mM tetranitroblue tetrazolium (Sigma, St. Louis, USA), 0.2 M sodium succinate,14 mM sodium azide, and 0.1 M sodium phosphate buffer, pH 7.6. Biopsy sections were incubated for 20 minutes in the dark before the reaction was briefly stopped in 0.01 M HCl, washed and mounted with glycerine gelatin. Samples were subsequently stored at 4^o^C and images were made within 10 days of staining using a light microscope (Leica DMRB, Wetzlar, Germany) using a 10x objective, and absorbance was measured by microdensitometry with an interference filter at 660 nm using Image J (National Institutes of Health, Bethesda, USA). Weighted average SDH activity was determined from at least 40 individual fibres from each participant (mean ± SD = 68 ± 23 per participant), with care taken to exclude fibres that were cut in a longitudinal orientation. Values were expressed as ΔA_660_ per µm tissue thickness per second of staining time (ΔA_660_·µm^-1^·s^-1^). For each participant, individual fibres were rank-ordered from highest to lowest for SDH activity. Subsequently, the 10 fibres with highest and lowest SDH activity for each subject were classified as high- and low-oxidative fibres, respectively.

*Capillarization*. Capillarization was assessed by staining for *Ulex Europaeus* Agglutin 1 lectin (UEA-1). Sections were air-dried for 10 minutes and then fixed in ice cold acetone (-20^o^C) for 15 minutes. Subsequently, slides were washed 3 times for 2 minutes in 1x PBS and blocked with 0.1% bovine serum albumin (SP-505 Vector Laboratories, Inc., Burlingame, CA, USA) for 30 minutes. Afterwards, slides were incubated with 20 µg/mL UEA-I (B-1065-2, Vector Laboratories) for 30 minutes at room temperature. Following this another washing step was performed, followed by incubation with VECTASTAIN ® Elite ABC-HRP Kit Peroxidase (PK-6100, Vector Laboratories) for 30 minutes at room temperature. Another washing step was performed, followed by incubation with ImmPACT™ AMEC Red Peroxidase Substrate (SK-4285, Vector Laboratories) for 10 minutes at room temperature. Finally, a washing step was performed in ddH_2_O for 5 minutes and sections were mounted with glycerine-gelatin (pre-warmed to 37^o^C). Capillary density (the number of capillaries per mm^2^) and the capillary-to-fibre ratio were calculated using ImageJ.

*Transmission electron microscopy*. Samples were fixed in 2.5% glutaraldehyde solution containing 0.1 M sodium cacodylate buffer (pH 7.3) for 24 h at room temperature and then transferred to storage buffer until later analysis. Samples were subsequently rinsed with ddH_2_O for 10 minutes, before post-fixation with 1% osmium tetroxide and 1.5% potassium ferrocyanide in ddH_2_O for 60 minutes at room temperature. Following post-fixation, samples were rinsed for 10 minutes with ddH_2_O, and dehydrated via a series of 15 minute incubations with a graded series of alcohol concentrations (i.e. 2x70, 80, 90% and 2x100% ethanol). Following dehydration, samples were impregnated with a 1:1 propylene oxide/Epon812 mixture for 1 h at room temperature, before being impregnated with Epon812 at 37^o^C for 30 min. Finally, samples were embedded longitudinally in moulds containing fresh Epon812 and left to polymerize for 3 days at 65^o^C. Ultra-thin (10 nm) longitudinal sections cut using a Leica Ultracut EM UC7 and collected on 150-mesh Formavar-coated copper grids and contrasted by staining for 5 min with 3.5% (w/v) uranyl acetate and 5 min with 3% (w/v) lead citrate. Visualisation was performed with a FEI Tecnai 120kV Transmission electron microscope (TEM, ThermoFisher Scientific, Waltman, Massachusetts, VS) using a Veleta camera Plus. Due to limited tissue availability, TEM data were only available for *n* = 9 young and *n* = 10 middle-aged participants. Four individual fibres were imaged per subject, with four images of the subsarcolemmal region and four images of the intermyofibrillar region being taken per fibre, resulting in approximately 16 images per subject per region, or 32 images across both regions per subject, for the analysis of individual mitochondrial area (in µm^2^), mitochondrial number (as number.µm^-2^), and mitochondrial area density (percentage muscle area occupied by mitochondria). Images were taken in a randomized, systematic fashion, at a magnification of 18,500x for quantification of mitochondrial area density and morphology, and at 48,500x for cristae density measurements, and analysed with ImageJ. Values were calculated for each fibre and averaged across fibres to create an average value for each participant. For mitochondrial area density, the summed mitochondrial area across the four fibres is presented as a percentage of the summed image area across the four fibres. The mitochondrial fragmentation index was calculated as the total mitochondrial number divided by the total mitochondrial area, and used as an indicator of the degree of mitochondrial fragmentation^6^. Morphological descriptors were also recorded, including: perimeter (µm), aspect ratio (i.e. the length-to-width ratio of a bounding box [major axis/minor axis]), maximal and minimal Ferret’s diameter (i.e. the longest and shortest distances, respectively, between two parallel tangents at opposing boundaries of a given mitochondria, µm), the perimeter-to-area ratio (i.e. the 2-dimensional measure that is analogous to the surface area-to-volume ratio of a 3-dimensional object), circularity (i.e. 4π [area/perimeter^2^], with a value of 1 indicating a perfect circle), and roundness (i.e. 4*area/[π*major axis^2^]). Mitochondrial cristae ultrastructure was determined by manually tracing the outlines of cristae in ImageJ. Eight randomly selected mitochondrial profiles were utilized per subject for this analysis^7^, with an even distribution between subsarcolemmal and intermyofibrillar mitochondrial profiles. The mitochondrial cristae area was expressed relative to mitochondrial area to yield cristae density. Cristae surface area density was previously calculated as the product of intermyofibrillar mitochondrial density and cristae density^8^. However, this method assumes that the overall mitochondrial density is similarly comprised between distinct populations. If, for two populations with the same mitochondrial density, there is a difference between them in terms of mean mitochondrial size and/or number per µm^2^, then cristae surface area density will be underestimated for the population with smaller and more numerous mitochondria, which will lead to an overestimation of respiration per cristae surface area. Hence, to calculate cristae surface area density in the present study, cristae density was multiplied by mitochondrial number per µm^2^ before multiplication by mitochondrial density. Finally, the images used for the cristae analysis were scored on a scale of 0 (no lipid, little glycogen) to 5 (extreme lipid and glycogen accumulation) for glycogen and intramyocellular lipid deposition by three independent raters in a blinded fashion, as previously described^9^.

*High-resolution respirometry*. Mitochondrial respiration was assessed in permeabilized skeletal muscle fibres as described previously^1^. Muscle fibres were dissected free of connective tissue, fat tissue and blood using sharp forceps. Small bundles of freshly isolated fibres were then placed in ice-cold relaxing buffer (BIOPS) containing 2.7 mM CaK_2_EGTA, 7.23 mM K_2_EGTA, 5.77 mM Na_2_ATP, 6.56 mM MgCl_2_·6H_2_O, 20mM taurine, 15mM Na_2_Phosphocreatine, 20mM imidazole, 0.5 mM dithiothreitol, 50 mM MES, with a pH of 7.1. Saponin (50 µg.mL^-1^) was then added to the BIOPS buffer to permeabilize the outer-cell membranes. After this, the fibres were transferred to respiration medium (MiR05) containing: 0.5 mM EGTA, 3.0 mM MgCl_2_·6H_2_O, 60 mM K-lactobionate, 20 mM taurine, 10 mM KH_2_PO_4_, 20 mM HEPES, 110 mM sucrose, 1 g.L^-1^ BSA, with pH of 7.1. Muscle fibres were blotted dry, weighed and transferred to a respirometer (Oxygraph-2k, Oroboros, Innsbruck, Austria) in respiration medium at 37^o^C. Oxygen concentration was between 300-500 µM throughout all experiments to avoid limitations in oxygen supply (i.e., an approximate PO_2_ of 175-300 mmHg). Background respiration was assessed before adding substrates and was subtracted from all subsequent values. Leak respiration was assessed after the addition of sodium glutamate (10 mM), sodium malate (0.5 mM), and sodium pyruvate (5 mM). NADH-linked respiration was assessed after the addition of 5 mM ADP and 10 mM cytochrome c to account for outer mitochondrial membrane integrity. No sample displayed an increase of greater than 10% following cytochrome c addition. Maximal oxidative phosphorylation (OXPHOS) capacity, with convergent electron input via complexes I and II, was assessed after the addition of 10 mM succinate. Electron transport system (ETS) capacity was determined via titration of carbonylcyanide-4-trifluoro- methoxyphenylhydrazone (FCCP) in 0.5 µM steps until no further increase in oxygen consumption was observed. Succinate-linked respiration (S+ROT) was measured following inhibition of mitochondrial complex I via the addition of 0.5 µM rotenone. Respiration experiments were performed in duplicate or triplicate depending on tissue availability, and the results were averaged. Respiration values were normalized to wet weight and expressed in pmol O_2_.s^-1^.mg^-1^.

Three separate approaches were employed to assess intrinsic mitochondrial respiration (i.e. mitochondrial quality), independent of mitochondrial abundance. Firstly, values for respiration in each respiratory state outlined above (i.e. leak, NADH-linked, OXPHOS, ETS, succinate-linked respiration) were normalized to the unique intermyofibrillar mitochondrial area density calculated from the TEM analysis for each individual. The second approach was to calculate various respiratory control ratios/factors from respiration values in the various respiratory states that were measured. As the measurements in each respiratory state are made in the same sample, and thus the same population of mitochondria, they can be utilized to provide insight into aspects of intrinsic mitochondrial respiratory function. The following respiratory control ratios were thus calculated: OXPHOS/ETS, LEAK/ETS, LEAK/OXPHOS, LEAK/NADH-linked, ROT+S/ETS, (OXPHOS – LEAK) / ETS, (OXPHOS – LEAK) / OXPHOS, (ETS – LEAK) / ETS, (ETS – OXPHOS) / ETS (see Figure S6 for a further description of these ratios). Finally, values for respiration in each respiratory state were normalized by the cristae surface area density in order to yield cristae specific respiration.

*Statistical analyses*. All data are presented as group means ± SD unless otherwise indicated. Data normality was tested using a Shapiro-Wilkes normality test. Differences between group means were assessed using independent samples t-tests when data were normally distributed and Mann-Whitney U test when non-normally distributed. Correlations between variables were assessed using Pearson’s correlation coefficient. Multiple linear regressions were used for variables hypothesized to influence $\dot{V}$O_2max_ and muscle O_2_ extraction. Any variable with a Variance Inflation Factor score > 5 was subsequently excluded from the model due to multicolinearity. Density distributions were obtained distribution using R studio with the ‘geom_density’ function of ggplot2 with a Gaussian kernel for density estimation. All other statistical analysis was performed using GraphPad Prism version 9 (GraphPad Software, La Jolla, CA). Statistical significance was accepted when *P* < 0.05.

**Supplementary Table 1.** Variables derived from cardiopulmonary exercise testing and physical activity monitoring. Values displayed are mean ± SD.

| **CPET variable** | **Young** | **Middle-aged** | **P value** |
| --- | --- | --- | --- |
| Peak power output (W) | 297 ± 53 | 247 ± 66 | 0.06 |
| Peak power output (W.kg^-1^) | 4.27 ± 0.50 | 3.10 ± 0.60 | <0.0001 |
| $\dot{V}$O_2max_ (L.min^-1^) | 3.15 ± 0.68 | 2.73 ± 0.65 | 0.16 |
| $\dot{V}$O_2max_ (mL.kg^-1^.min^-1^) | 45.2 ± 7.0 | 34.4 ± 5.4 | <0.0001 |
| Gain of $\dot{V}$O_2_ vs. power output (mL.W^-1^.min^-1^) | 8.38 ± 1.02 | 8.89 ± 1.31 | 0.31 |
| GET (L.min^-1^) | 1.71 ± 1.12 | 1.43 ± 1.09 | 0.56 |
| GET (mL.kg^-1^.min^-1^) | 32.1 ± 3.9 | 24.4 ± 5.1 | 0.0006 |
| GET (%$\dot{V}$O_2max_) | 71.5 ± 6.5 | 70.6 ± 6.0 | 0.73 |
| RCP (L.min^-1^) | 2.10 ± 1.35 | 1.78 ± 1.34 | 0.57 |
| RCP (mL.kg^-1^.min^-1^) | 39.8 ± 3.6 | 29.5 ± 5.0 | <0.0001 |
| RCP (%$\dot{V}$O_2max_) | 89 ± 8 | 89 ± 7 | 0.93 |
| HR_max_ (beats.min^-1^) | 188 ± 7 | 171 ± 7 | <0.0001 |
| $\dot{V}$O_2_-HR slope (beats.L^-1^.min^-1^) | 32 ± 12 | 34 ± 15 | 0.82 |
| $\dot{V}$E-$\dot{V}$CO_2_ slope | 26.1 ± 3.4 | 27.7 ± 5.7 | 0.42 |
| $\dot{V}$CO_2max_ (L.min^-1^) | 3.92 ± 0.90 | 3.30 ± 0.75 | 0.096 |
| O_2_ pulse_max_ (mL.beat^-1^) | 17.1 ± 3.5 | 16.7 ± 4.2 | 0.79 |
| RER_max_ | 1.20 ± 0.19 | 1.24 ± 0.07 | 0.54 |
| $\dot{V}$E_max_ (L.min^-1^) | 138.28 ± 30.97 | 116.21 ± 39.40 | 0.16 |
| $\dot{V}$E/$\dot{V}$O_2max_ | 44.2 ± 4.5 | 42.6 ± 5.6 | 0.46 |
| $\dot{V}$E/$\dot{V}$CO_2max_ | 26.4 ± 4.7 | 28.9 ± 5.8 | 0.28 |
| PETO_2max_ (mmHg) | 123.0 ± 2.7 | 120.9 ± 4.0 | 0.16 |
| PETCO_2max_ (mmHg) | 45.7 ± 4.5 | 44.3 ± 4.4 | 0.47 |
| VT_max_ (L) | 2.9 ± 0.8 | 3.1 ± 0.6 | 0.60 |
| B_Fmax_ (breaths.min^-1^) | 57 ± 9 | 46 ± 11 | 0.020 |
| Daily steps (count, n=10) | 6540 ± 1769 | 7464 ± 3293 | 0.64 |
| Moderate physical activity (min.week^-1^, n=12) | 227 ± 274 | 161 ± 93 | 0.98 |
| Intense physical activity (min.week^-1^, n=12) | 232 ± 78 | 205 ± 49 | 0.46 |
| CPET: cardiopulmonary exercise test; $\dot{V}$O_2max_, maximal oxygen uptake; GET, gas exchange threshold; RCP, respiratory compensation point; HR, heart rate; $\dot{V}$E, ventilation; $\dot{V}$CO_2_, carbon dioxide output; RER, respiratory exchange ratio; PETO_2max_, end-tidal pressure of O_2_ at maximal exercise; PETCO_2max_, end-tidal pressure of CO_2_ at maximal exercise; VT_max_, tidal volume at maximal exercise; B_F_, breathing frequency. | | | |

**Supplementary Figures**


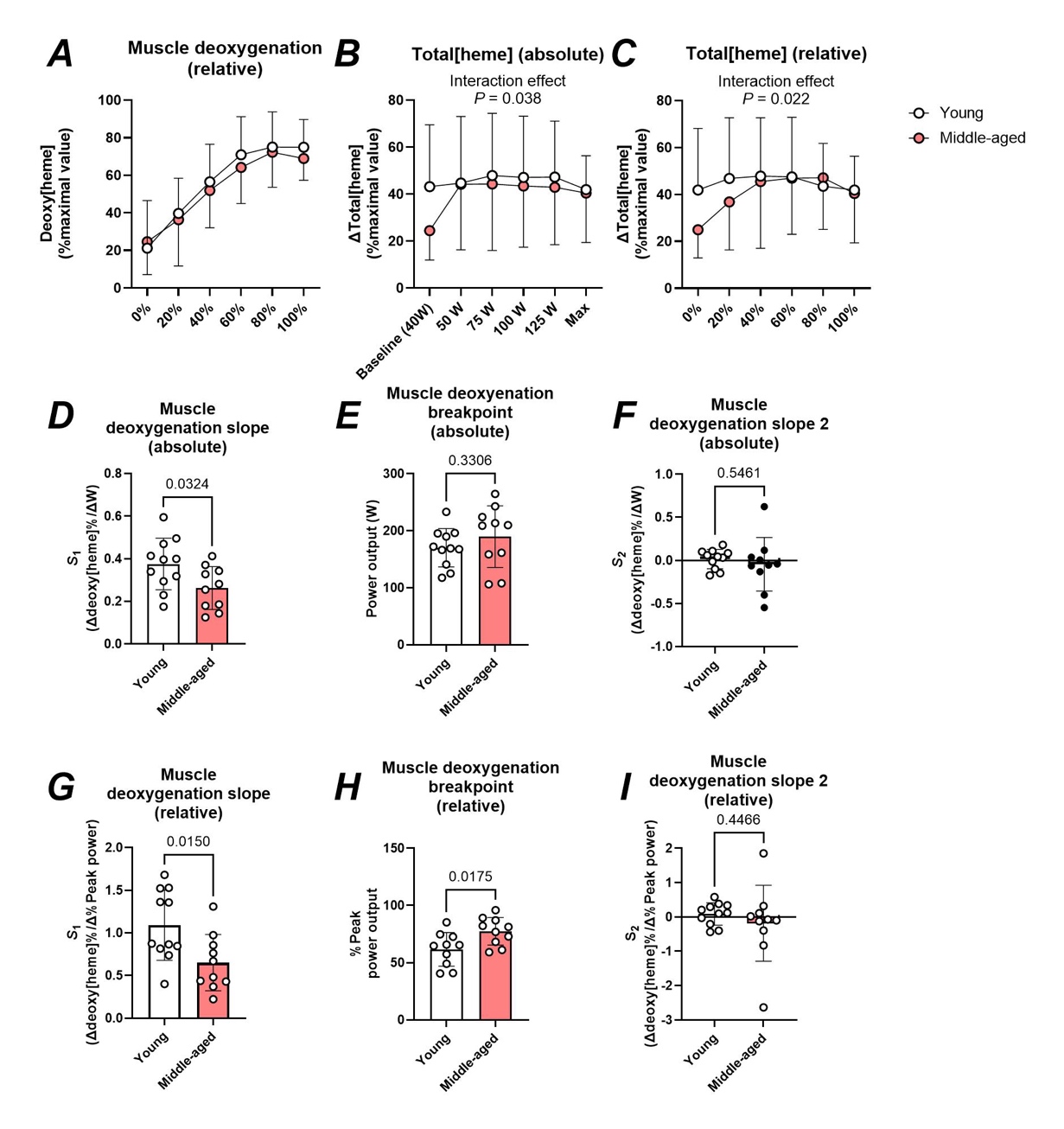


**Supplemental Figure 1. Blunted muscle O_2_ extraction in middle-age humans during exercise.** Muscle O_2_ extraction and capillary [hematocrit] changes during exercise were assessed via the deoxy[heme] and total[heme] signals derived from near-infrared spectroscopy (NIRS), respectively. Group mean ± SD deoxy[heme] (***A***) and total[heme] (***B*** & ***C***) responses to the incremental exercise protocol are displayed in the top panels as a function of absolute (***B***) and relative (***A*** & ***C***) work rate. Muscle deoxy[heme] responses to incremental exercise were fitted with a double linear function to characterize O_2_ extraction during exercise. The fit is described by an initial slope (***D*** & ***G***), a breakpoint (***E*** & ***H***), and a second slope (***F*** & ***I***). Responses were fitted twice, as a function of absolute power output (***D***-***F***) and as a percentage of the peak power output attained during the incremental test (i.e., relative power output, ***G***-***I***). Circles reflect individual data points for each participant. Columns and error bars reflect means ± SD. Data in panels ***F*** and ***I*** were analysed using a Mann-Whitney U test, all other data were analysed via independent samples t-tests. 11 young participants and 10 middle-aged participants were included for each comparison. *P* values are displayed above each panel.


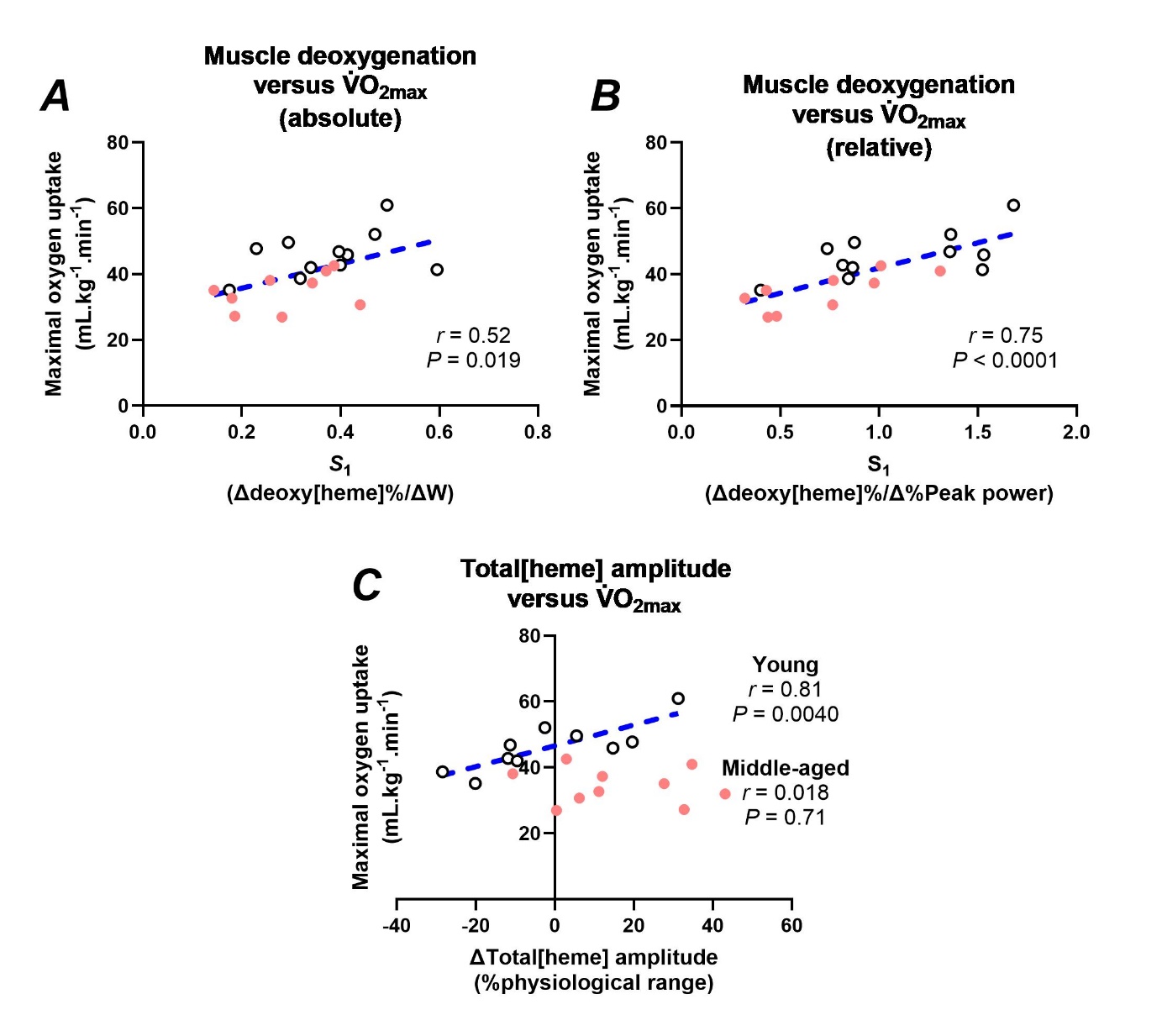


**Supplementary Figure 2. Muscle O_2_ extraction is positively related to maximal oxygen uptake (**$\dot{\boldsymbol{V}}$**O_2max_).** The slopes of muscle deoxy[heme] against absolute (***A***) and relative (***B***) power output throughout the initial portion of the incremental test were positively correlated with $\dot{V}$O_2max_. The amplitude of change in total[heme] from rest to maximal exercise was positively correlated with $\dot{V}$O_2max_ in young but not middle-aged participants (**C**). Filled circles represent middle-aged subjects, open circles represent young individuals. Solid blue line indicates line of best fit. 11 young participants and 10 middle-aged participants were included for each comparison. Two-sided Pearson’s correlations were performed in both cases. *R* and *P* values are displayed on the inset of each panel.


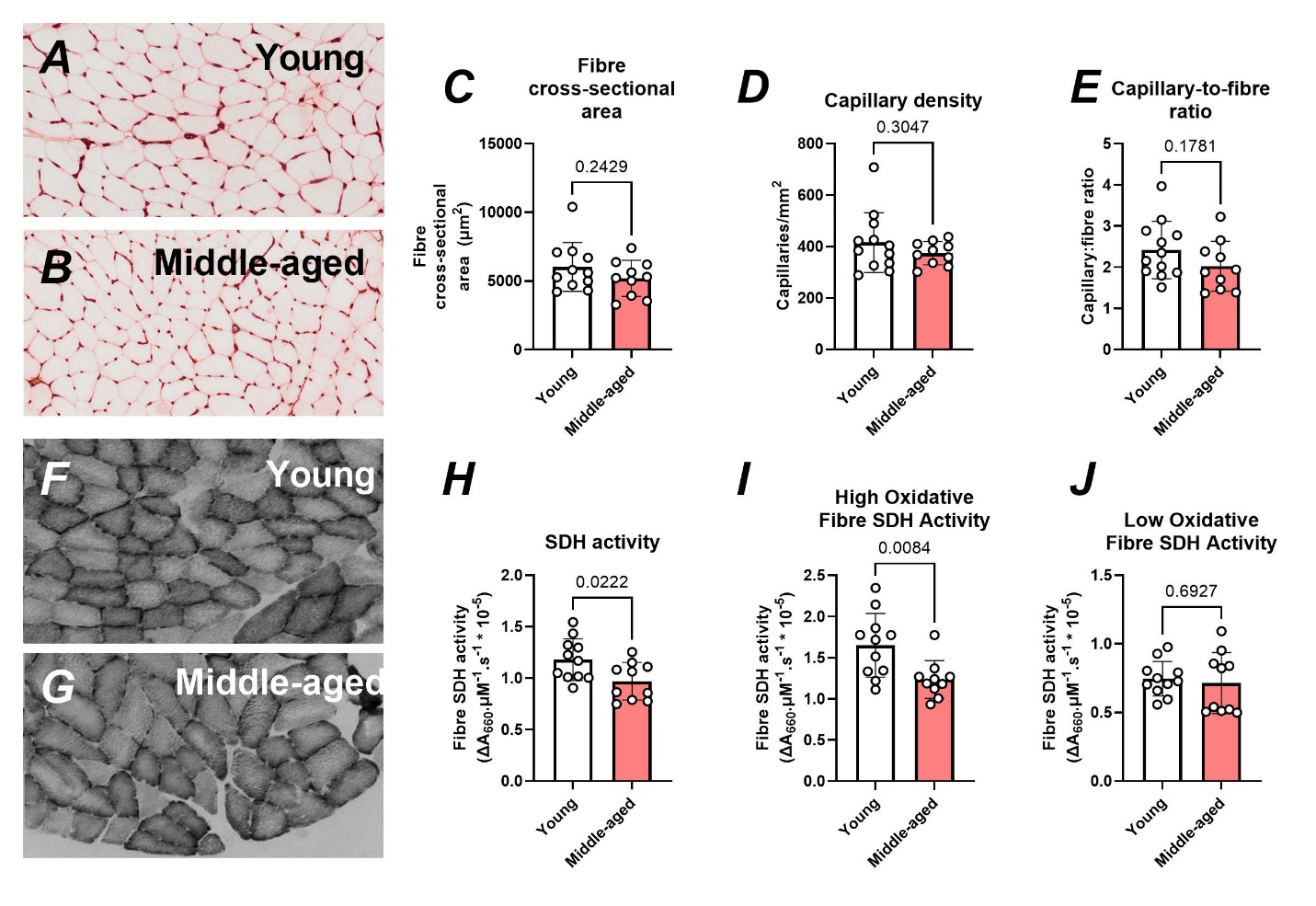


**Supplementary Figure 3. Reduced physical capacity in middle-age is related to lower mitochondrial enzyme activity rather than muscle capillarization.** We stained 10 µm thick frozen muscle sections for capillaries (***A*** & ***B***) and succinate dehydrogenase (SDH) activity (***F*** & ***G***). Despite the age difference between groups, no differences in mean muscle fibre cross-sectional area were observed (***C***, n=11 young, n=10 middle-aged). Similarly, no differences were observed regarding capillary density (***D***, n=12 young, n=10 middle-aged) or the capillary-to-fibre ratio (***E***, n=12 young, n=10 middle-aged). Mean fibre SDH activity was lower in the middle-aged group (***H***, n=11 young, n=10 middle-aged), an effect which appeared to be driven by the most (***I***, n=11 young, n=10 middle-aged) as opposed to the least (***J***, n=11 young, n=10 middle-aged) oxidative fibres. Circles reflect individual data points for each participant. Columns and error bars reflect means ± SD. Data in panel ***D*** were analysed using a Mann-Whitney U test, all other data were analysed via independent samples t-tests. *P* values are displayed above each panel.


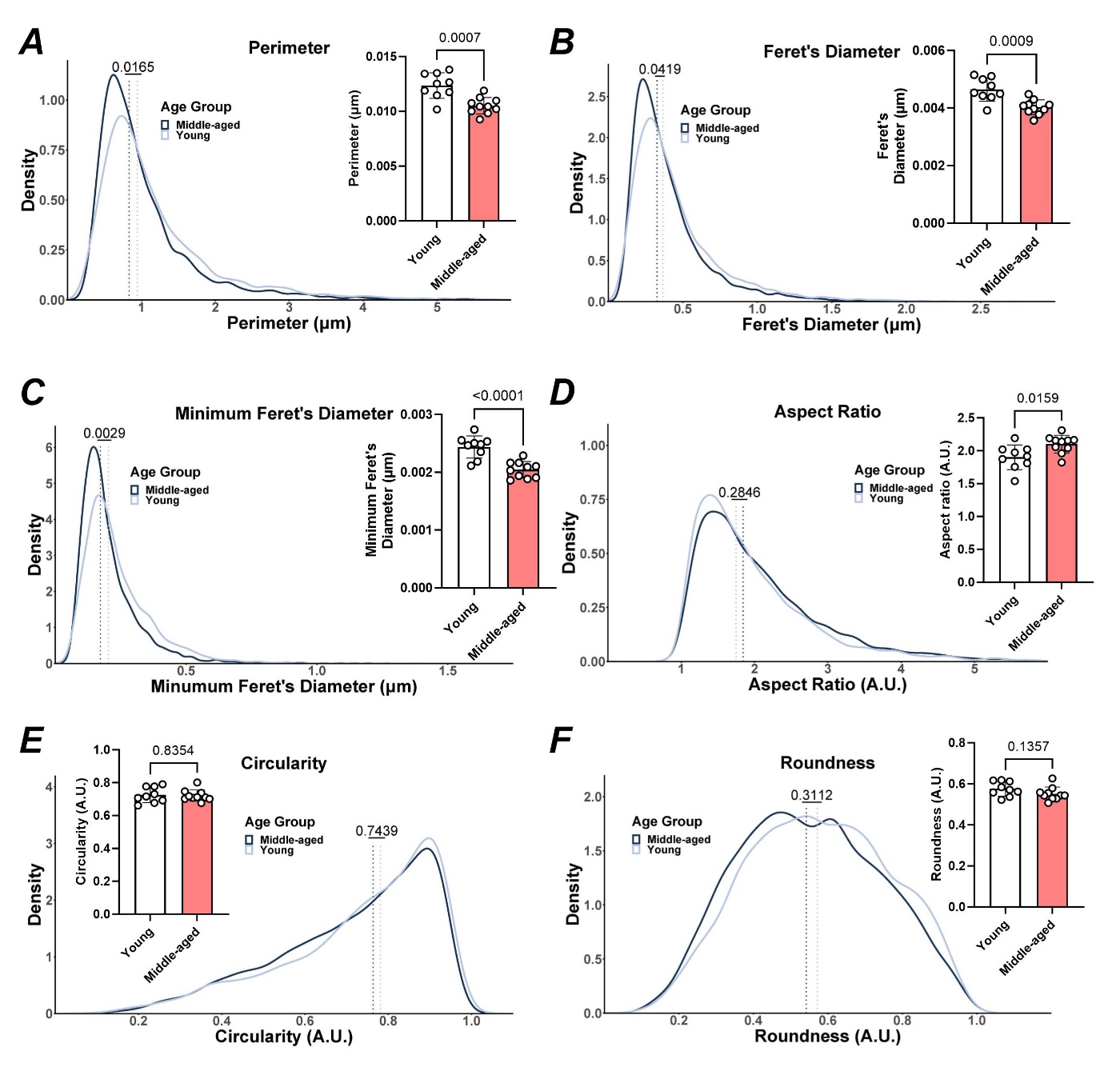


**Supplementary Figure 4. Ageing affects skeletal muscle intermyofibrillar mitochondrial size but not shape descriptors.** Transmission electron microscopy was performed on single skeletal muscle fibres obtained via muscle biopsy. The morphology of muscle mitochondria was assessed by manually tracing the outlines of 8,025 intermyofibrillar mitochondrial profiles. The middle-aged individuals displayed reductions in morphological descriptors relating to mitochondrial size (i.e., ***A***, perimeter; ***B***, maximum; and ***C***, minimum Feret’s diameter), but not those pertaining to shape (i.e., ***D***, aspect ratio; ***E***, circularity; ***F***, roundness). Density distributions are displayed as light (young) and dark blue (middle-aged) curves, with dashed lines indicating the median value for each distribution. *P* values from each independent t-test are displayed above each distribution. Group mean ± SD data (columns and error bars) along with individual mean values (open circles) for each subject displayed on the inset of each panel. *P* values are displayed for group comparisons above the columns on each inset panel.


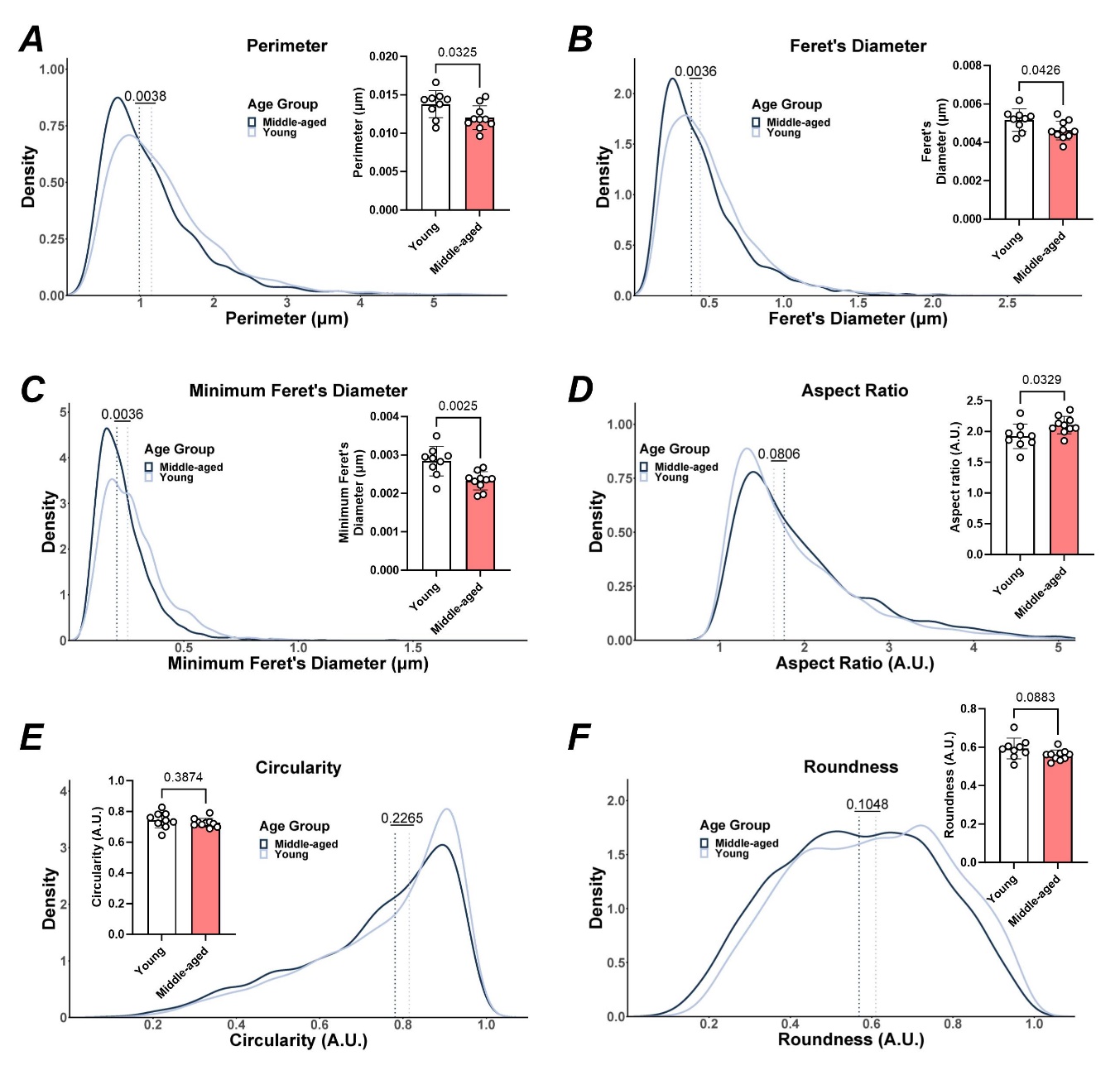


**Supplementary Figure 5. Ageing affects skeletal muscle subsarcolemmal mitochondrial size and aspect ratio.** Transmission electron microscopy was performed on single skeletal muscle fibres obtained via muscle biopsy. The morphology of muscle mitochondria was assessed by manually tracing the outlines of 6,651 subsarcolemmal mitochondrial profiles. ***A***, mitochondrial perimeter; ***B***, Feret’s diameter; ***C***, minimum Feret’s diameter; ***D***, mitochondrial aspect ratio; ***E***, circularity; ***F***, roundness. Density distributions are displayed as light (young) and dark blue (middle-aged) curves, with dashed lines indicating the median value for each distribution. P values from each independent t-test are displayed above each distribution. Group mean ± SD data (columns and error bars) along with individual mean values (open circles) for each subject displayed on the inset of each panel. *P* values are displayed for group comparisons above the columns on each inset panel.


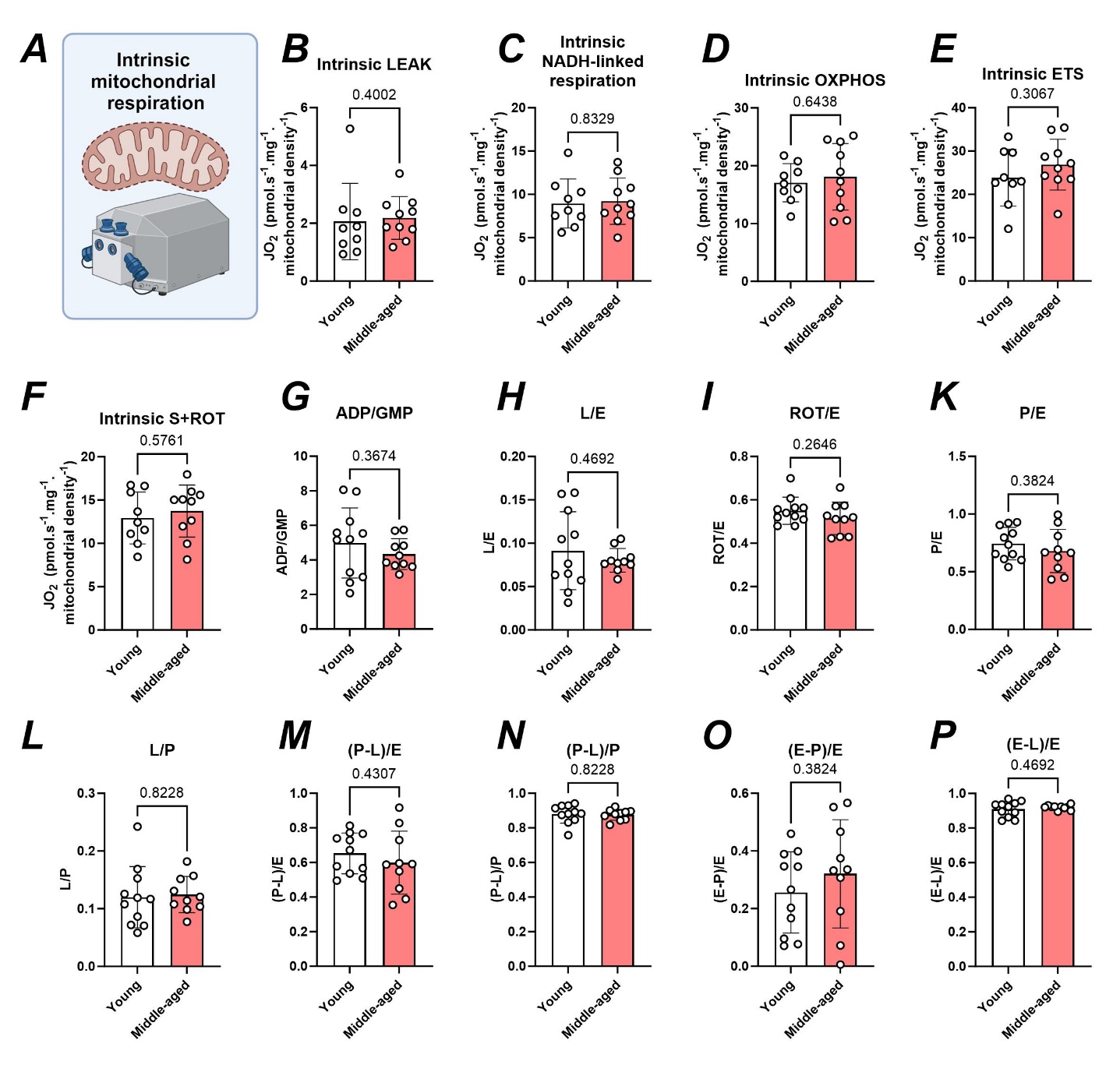


**Supplementary Figure 6. No age-related differences in intrinsic mitochondrial respiration**. We normalized mitochondrial respiration (Figure 3) by intermyofibrillar mitochondrial density to obtain intrinsic mitochondrial respiration, and calculated flux control ratios to provide an alternate indicator of intrinsic respiration (***A***). ***B***-***F***, values displayed are *J*O_2_ (oxygen flux per unit wet weight) normalized to intermyofibrillar mitochondrial area density. Group mean ± SD (columns and error bars) and individual data (open circles) are displayed in various respiratory states both in young and middle-aged groups. ***B***, LEAK respiration with NADH-linked substrates (i.e. pyruvate, malate and glutamate) in the absence of ADP (n=9 young, n=10 middle-aged); ***C***, respiration with NADH-linked substrates and saturating [ADP] (n=9 young, n=10 middle-aged); ***D***, maximal oxidative phosphorylation capacity with NADH-linked substrates and convergent electron flow through complex II via the addition of succinate (n=9 young, n=10 middle-aged); ***E***, maximal uncoupled respiration via sequential titrations of uncoupler to yield electron transport system (ETS) capacity (n=9 young, n=10 middle-aged); ***F***, uncoupled respiration following inhibition of mitochondrial complex I via the addition of rotenone (S+ROT) (n=9 young, n=10 middle-aged). ***G***-***P***, values displayed are arbitrary ratios of respiratory states measured with different substrate combinations (n=11 young, n=10 middle-aged for each comparison). GMP, glutamate, malate and pyruvate; ADP, NADH-linked respiration, ROT, uncoupled respiration with rotenone and succinate; E, maximal uncoupled respiration with optimal concentrations of uncoupler and convergent electron flow through complexes I and II; L, leak respiration with NADH-linked substrates (i.e. pyruvate, malate and glutamate) in the absence of ADP; P, maximal phosphorylating respiration with NADH-linked substrates (i.e. pyruvate, malate and glutamate), succinate, and saturating concentrations of ADP. Data in panel ***B*** were analysed using a Mann-Whitney U test, all other data were analysed via independent samples t-tests. *P* values are displayed above each panel.


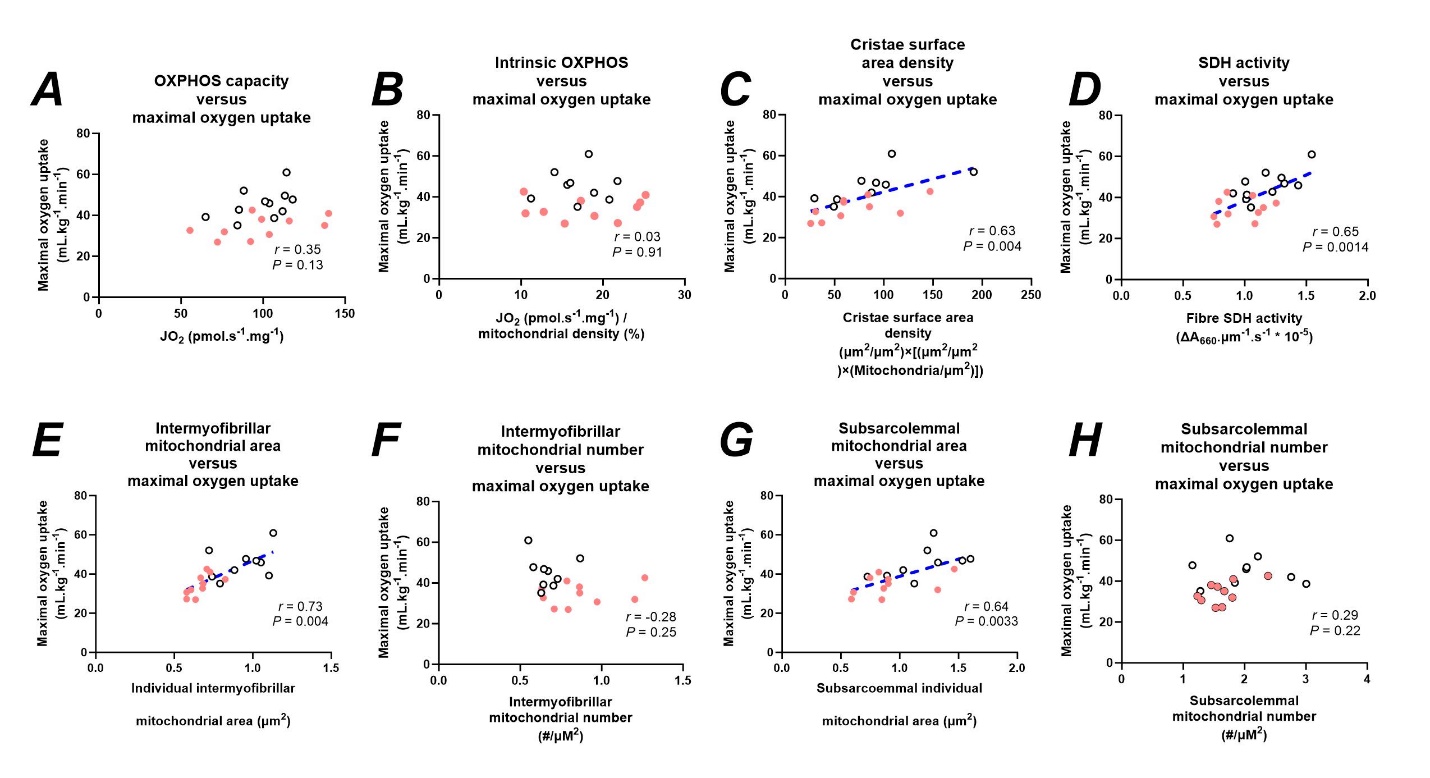


**Supplementary Figure 7. Relationships between mitochondrial variables and maximal oxygen uptake (**$\dot{\boldsymbol{V}}$**O_2max_).** In order to determine which variables contributed towards the age-associated reduction in $\dot{V}$O_2max_, we performed correlation analyses with the mitochondrial variables that were measured in the present study. ***A***, oxidative phosphorylation capacity (OXPHOS) (n=11 young, n=10 middle-aged); ***B***, oxidative phosphorylation capacity normalized by intermyofibrillar mitochondrial density (intrinsic OXPHOS) (n=9 young, n=10 middle-aged); ***C***, cristae surface area density (n=9 young, n=10 middle-aged); ***D***, succinate dehydrogenase (SDH) activity (n=11 young, n=10 middle-aged); ***E***, mean individual intermyofibrillar mitochondrial area (n=9 young, n=10 middle-aged); ***F***, mean intermyofibrillar mitochondrial number (n=9 young, n=10 middle-aged); ***G***, mean individual subsarcolemmal mitochondrial area (n=9 young, n=10 middle-aged); ***H***, mean subsarcolemmal mitochondrial number (n=9 young, n=10 middle-aged). Filled circles represent middle-aged participants whereas clear circles reflect young participants. Two-sided Pearson’s correlations were performed in each case. *R* and *P* values are displayed on the inset of each panel.


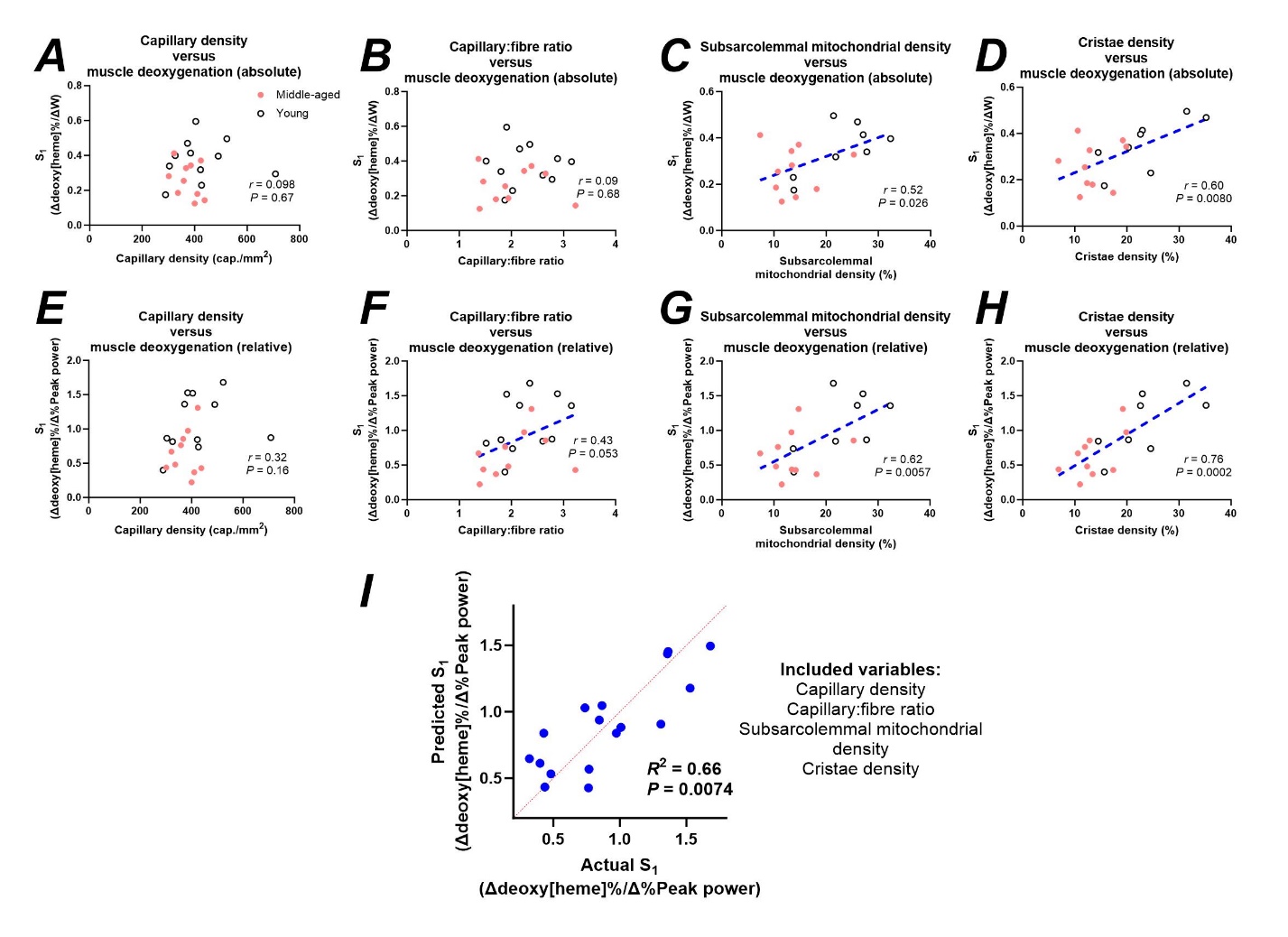


**Supplementary Figure 8. Muscle O_2_ extraction is more closely related to mitochondrial variables than capillarization.** Correlation analyses were performed relating the slope of muscle deoxy[heme] during incremental exercise against absolute (***A***-***D***) and relative (***E***-***H***) power output versus variables hypothesized to influence muscle O_2_ extraction. Generally, variables related to muscle mitochondria were more strongly correlated to muscle O_2_ extraction than those related to capillarization. ***A*** & ***E***, capillary density (n=11 young, n=10 middle-aged for both comparisons); ***B*** & ***F***, capillary-to-fibre ratio (n=11 young, n=10 middle-aged for both comparisons); ***C*** & ***G***, subsarcolemmal mitochondrial density (n=8 young, n=10 middle-aged for both comparisons); ***D*** & ***H***, cristae density (n=8 young, n=10 middle-aged for both comparisons). Filled circles represent middle-aged subjects, open circles represent young individuals. Dashed blue line indicates line of best fit. Two-sided Pearson’s correlations were performed. *R* and *P* values are displayed on the inset of each panel. ***I***, multiple linear regression of variables hypothesized to influence muscle O_2_ extraction (capillary density, capillary-to-fibre ratio, subsarcolemmal mitochondrial density, cristae density) with the initial slope of muscle deoxy[heme] (S_1_) against relative power output (n=8 young, n=10 middle-aged for both comparisons). The included variables explained 66% of variance associated with S_1_ (*P* = 0.0074). The highest variance inflation factor score was 2.081. Blue circles represent individual data points, dashed red line reflects the line of identity.


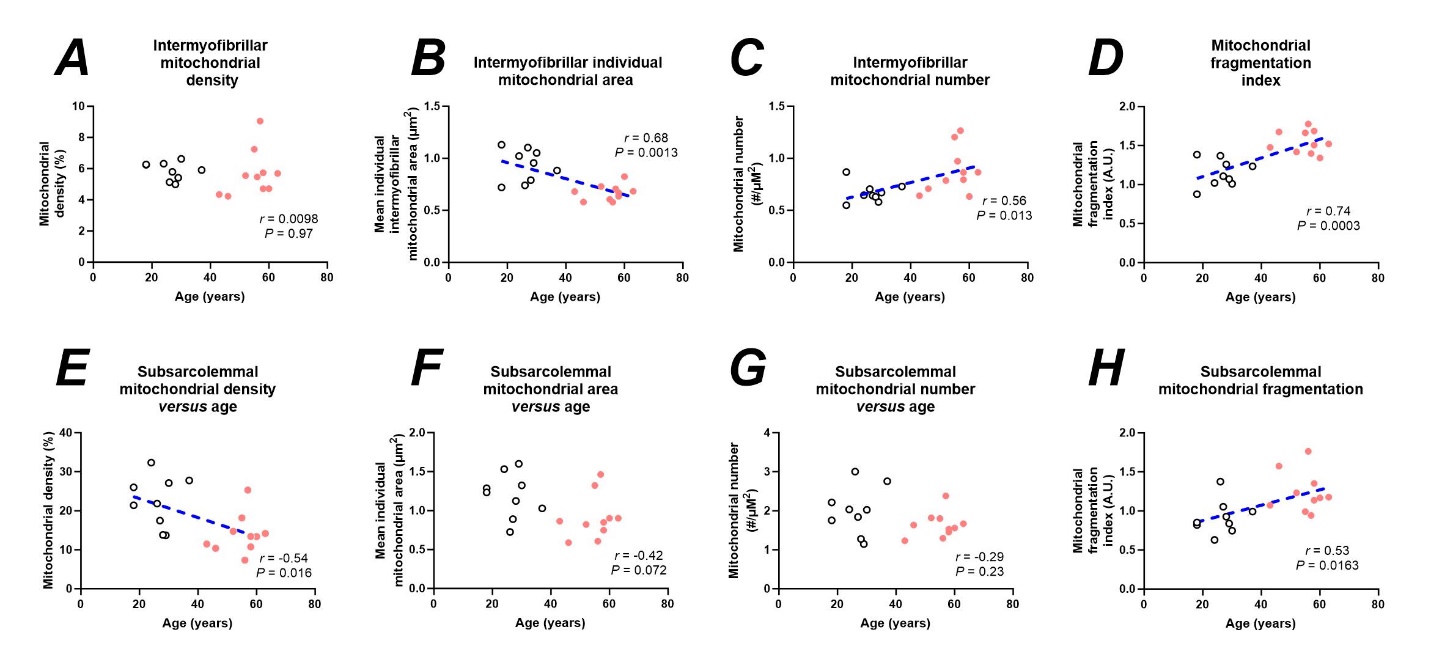


**Supplementary Figure 9**. **Progressive mitochondrial fragmentation with ageing.** Correlation analyses between mitochondrial variables and age were performed to determine the association between ageing and mitochondrial density, morphology, and fragmentation. Correlations for the intermyofibrillar region are displayed in panels ***A***-***D*** and correlations for the subsarcolemmal region are displayed in panels ***E***-***H***. ***A*** & ***E***, mitochondrial density; ***B*** & ***F***, mean individual mitochondrial area; ***C*** & ***G***, mean mitochondrial number per µm^2^; ***D*** & ***H***, mitochondrial fragmentation index. Filled circles represent middle-aged subjects, open circles represent young individuals. Dashed blue line indicates line of best fit. Two-sided Pearson’s correlations were performed for n=9 young and n=10 middle-aged participants in every case. *R* and *P* values are displayed on the inset of each panel.


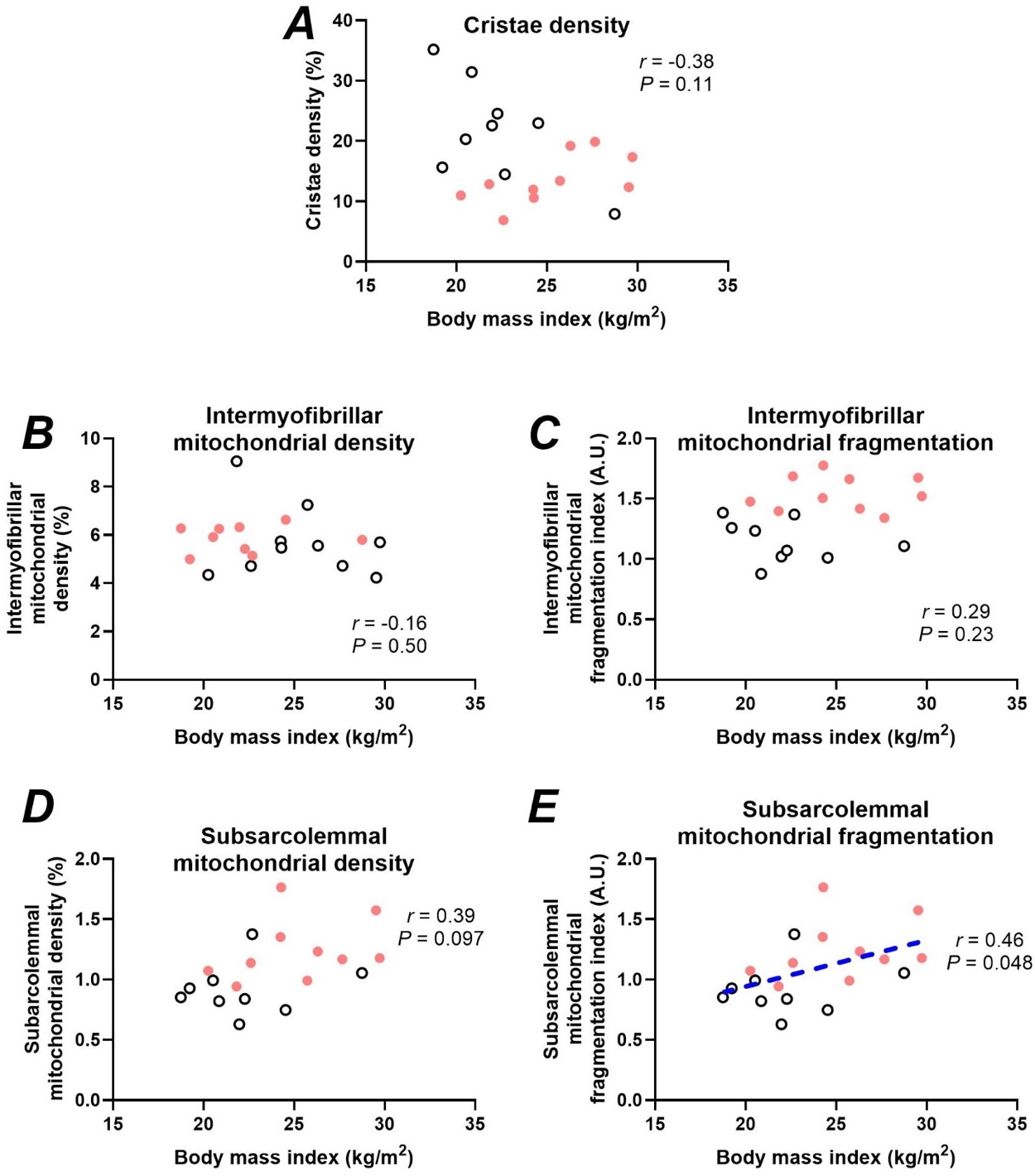


**Supplementary Figure 10. Body mass index is not significantly related to age-related skeletal muscle mitochondrial changes.** To determine whether the age-related skeletal muscle mitochondrial changes that we observed herein where due in part to the increased adiposity that is often associated with age, we performed correlations between body mass index (BMI) and the mitochondrial variables that displayed the most significant age-related changes in the present study: mitochondrial cristae density (***A***), mitochondrial density (***B***, intermyofibrillar; ***D***, subsarcolemmal), and mitochondrial fragmentation (***C***, intermyofibrillar; ***E***, subsarcolemmal). Filled circles represent middle-aged subjects, open circles represent young individuals. Blue dashed lines represent lines of best-fit. Two-sided Pearson’s correlations were performed for n=9 young and n=10 middle-aged participants in every case. *R* and *P* values are displayed on the inset of each panel.

**References**

1. Appelman, B. *et al.* Muscle abnormalities worsen after post-exertional malaise in long COVID. *Nat Commun* **15**, 17 (2024).

2. Poole, D. C. & Jones, A. M. Measurement of the maximum oxygen uptake V̇o2max: V̇o2peak is no longer acceptable. *J. Appl. Physiol.* **122**, 997–1002 (2017).

3. Goulding, R. P. *et al.* Limitations to exercise tolerance in type 1 diabetes: the role of pulmonary oxygen uptake kinetics and priming exercise. *J. Appl. Physiol.* **128 (5)**, 1299-1309 (2020).

4. van der Zwaard, S. *et al.* Maximal oxygen uptake is proportional to muscle fiber oxidative capacity, from chronic heart failure patients to professional cyclists. *J. Appl. Physiol.* **121**, 636–645 (2016).

5. Goulding, R. P. *et al.* Dissociation between exercise intensity thresholds: mechanistic insights from supine exercise. *Am J Physiol-Regul Integ Comp Physiol* **321**, R712–R722 (2021).

6. Gemmink, A. *et al.* Twenty-four hour rhythmicity in mitochondrial network connectivity and mitochondrial respiration; a study in human skeletal muscle biopsies of young lean and older individuals with obesity. *Mol Metab* **72**, 101727 (2023).

7. Nielsen, J. *et al.* Plasticity in mitochondrial cristae density allows metabolic capacity modulation in human skeletal muscle. *J Physiol* **595**, 2839–2847 (2017).

8. Schytz, C. T. *et al.* Skeletal muscle mitochondria demonstrate similar respiration per cristae surface area independent of training status and sex in healthy humans. *J Physiol* **602**, 129–151 (2024).

9. Eggelbusch, M. *et al.* The impact of bed rest on human skeletal muscle metabolism. *CR Med* **5 (1)**, 101372 (2024).
